# Unlocking cardiac health: exploring the role of class I HDACs in cardiovascular diseases

**DOI:** 10.1007/s11010-025-05353-5

**Published:** 2025-07-14

**Authors:** Padmini Pai, Rachel Savio D’Mello, Ojasvi Mangesh Brahme, Yoga Varshitha Gogineni, Manasa Gangadhar Shetty, Babitha Kampa Sundara

**Affiliations:** 1 Department of Biophysics, Manipal School of Life Sciences, Manipal Academy of Higher Education, Manipal, Karnataka 576104 India; 2 Present Address: Manipal School of Life Sciences, Manipal Academy of Higher Education, Manipal, Karnataka 576104 India

**Keywords:** Cardiac hypertrophy, Cardiovasular disease, Class I HDACs, Histone Deacetylase, Inhibitors

## Abstract

Translation modifications, particularly those involving histone deacetylases (HDACs), are crucial for regulating gene expression and have significant implications for cardiovascular disease (CVD). The association between HDACs and CVD has garnered significant research interest due to the pivotal role of HDACs in gene expression regulation and cellular function. Aberrant HDAC activity has been implicated in various pathological processes underlying CVD, including inflammation, endothelial dysfunction, hypertrophy, fibrosis, and atherosclerosis. This review explores the molecular mechanisms by which different HDAC isoforms influence cardiovascular pathology, highlighting evidence from preclinical and clinical studies. Emerging data suggest that HDAC inhibitors could offer therapeutic potential by modulating these processes and improving cardiovascular health. However, the precise isoform-specific roles and therapeutic window for HDAC inhibition in CVD remain to be elucidated. Furthermore, research is essential to identify the crucial and complex relationship between HDACs and cardiovascular health, which could pave the way for novel, targeted interventions for the treatment of CVD.

**Graphical abstract:**

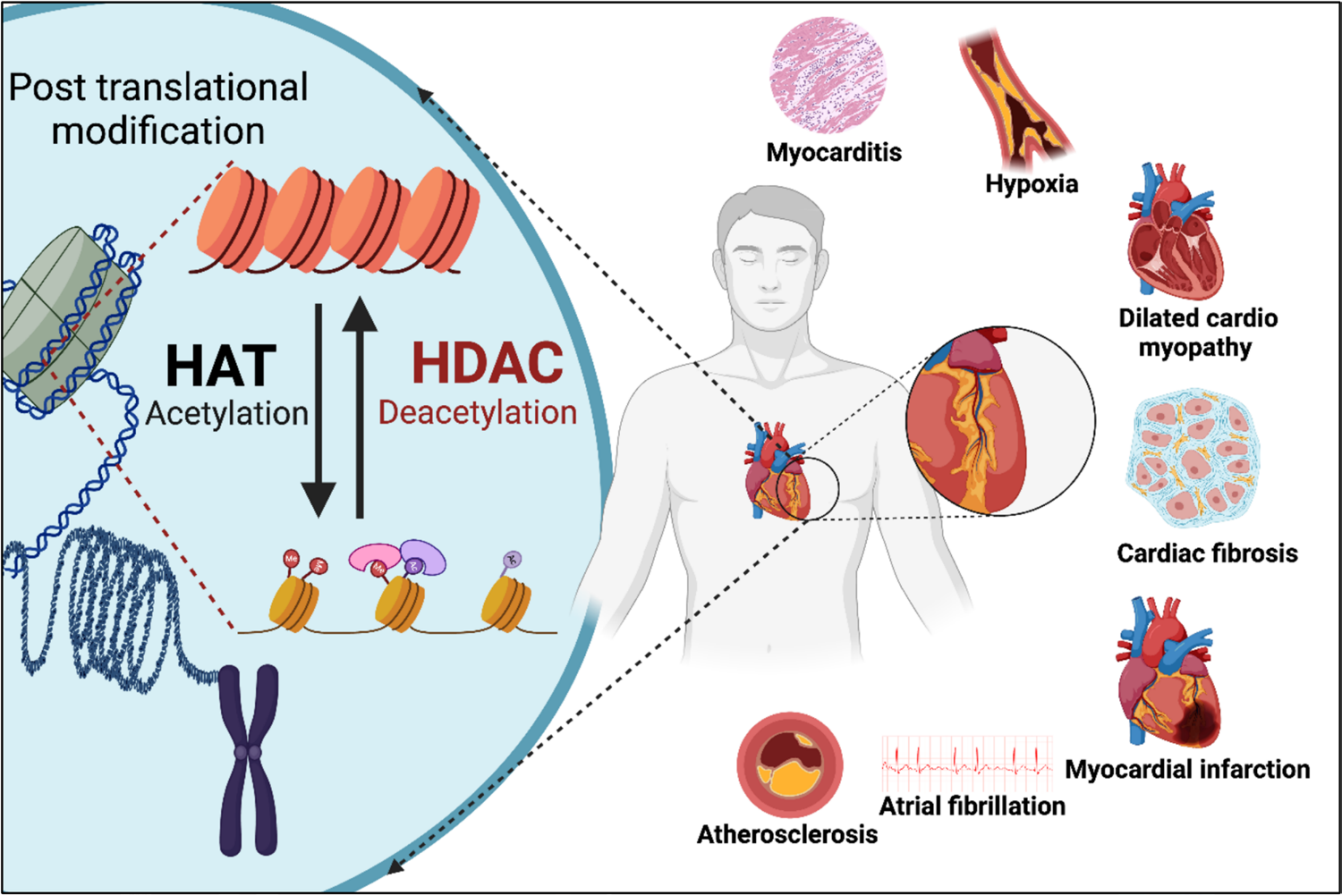

## Introduction

Cardiovascular disease (CVD) is common, with approximately 17.9 million deaths annually and is the leading cause of mortality and morbidity in developed countries. CVDs lead to an increasing loss of cardiomyocytes due to apoptosis or necrosis, which may culminate in cardiac dysfunction and ultimately death [1] The various cardiac cells, includes cardiomyocytes, cardiac fibroblasts, ventricular smooth muscle cells and endothelial cells ensure the smooth functioning of the heart [2]. Maintaining cardiac health involves supporting the integrity and function of cardiac cells through lifestyle choices and medical interventions. The key measures include regular exercise, avoiding smoking, adhering to a healthy diet, and managing conditions like hypertension and diabetes [3]. In addition, certain internal factors such as hypertension, high cholesterol, diabetes, etc., often inherited and epigenetic in nature, can lead to heart problems [4, 5].

**Fig. 1:**
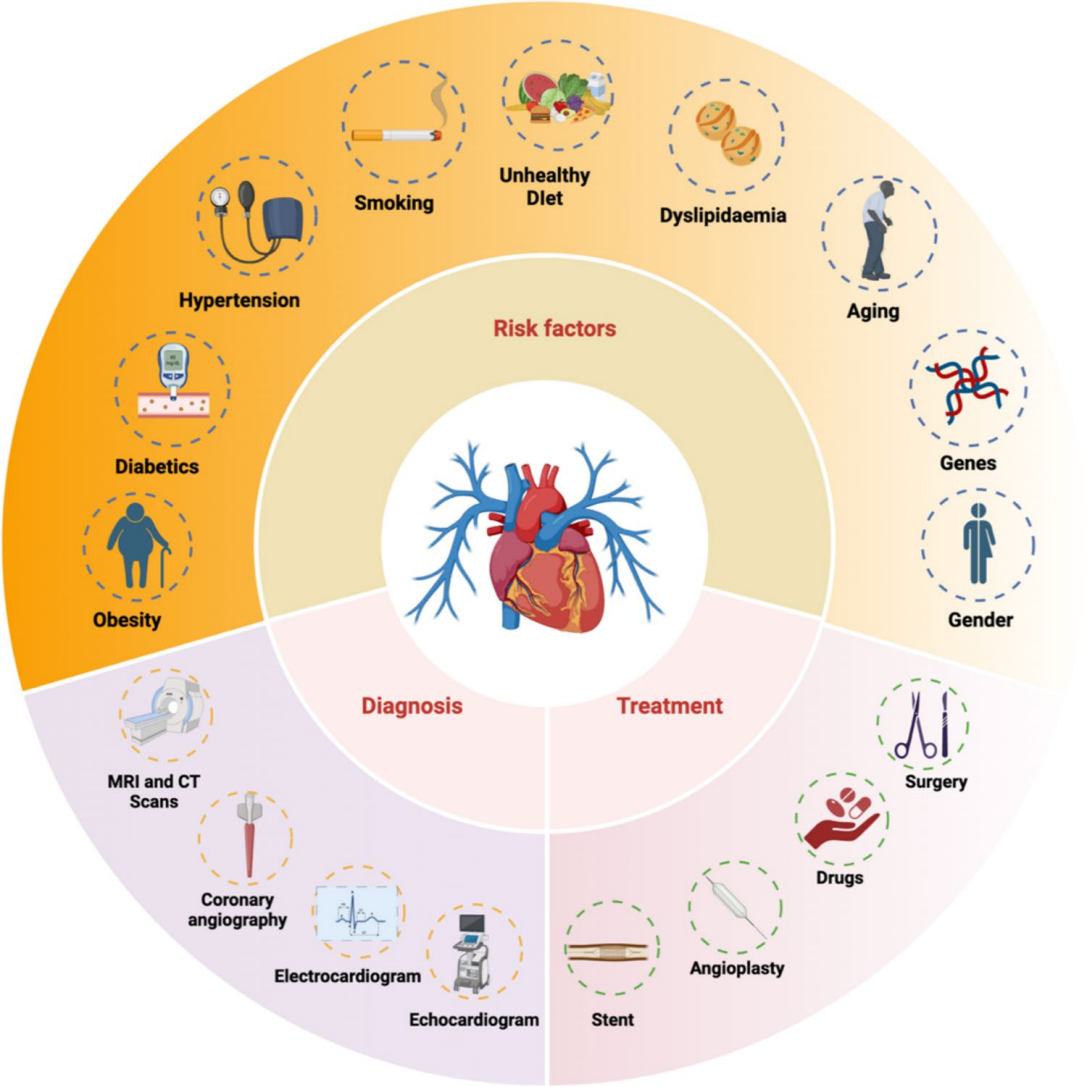
Risk factors, diagnosis and treatments of cardiovascular diseases

One of the prevalent CVDs is cardiac hypertrophy, which is caused by the unusual thickening of the heart wall muscles [6]. Cardiac hypertrophy often leads to cardiac fibrosis, which is the accumulation of excess amounts of fibrotic components such as collagen, extracellular matrix and other proteins in the heart, thus causing stiffening of the heart and leading to heart failure [7]. Atrial fibrillation, marked by abnormal bombardment of electrical impulses in atria that overrule the heart’s natural pacemaker, is another significant cardiovascular disorder [8]. The accumulation of plaque in the arterial walls of the heart, also known as atherosclerosis, is known to cause myocardial infarction (MI) during heart attack. This occurs due to a reduction in blood flow to the heart, a condition known as hypoxia [9]. The risk factors, diagnoses and treatments available for cardiovascular diseases are given in Fig. 1.

Histone deacetylases (HDACs) play a significant role in regulating transcription and gene expression in cardiomyocytes through various mechanisms, particularly by modulating inflammation in the heart. The 18 different mammalian HDACs are grouped into 4 different classes, each encoded by distinct genes [10]. These classes are defined based on their sequence motifs and catalytic action. Class I HDACs are composed of HDAC1, 2, 3 and 8 isoforms, which are characterized by a deacetylase domain [11]. Class II HDACs are further subdivided into IIa (HDAC4, 5, 7 and 9) and IIb (HDAC6 and 10) HDACs. Class III HDACs, known as sirtuins (SIRT1–7), require NAD^+^ for their activity and are involved in aging, metabolism, and stress responses. Class IV HDACs, represented solely by HDAC11, have common features with both Class I and II HDACs, and they play important roles in immune regulation and metabolic processes in mice [12].

**Fig. 2:**
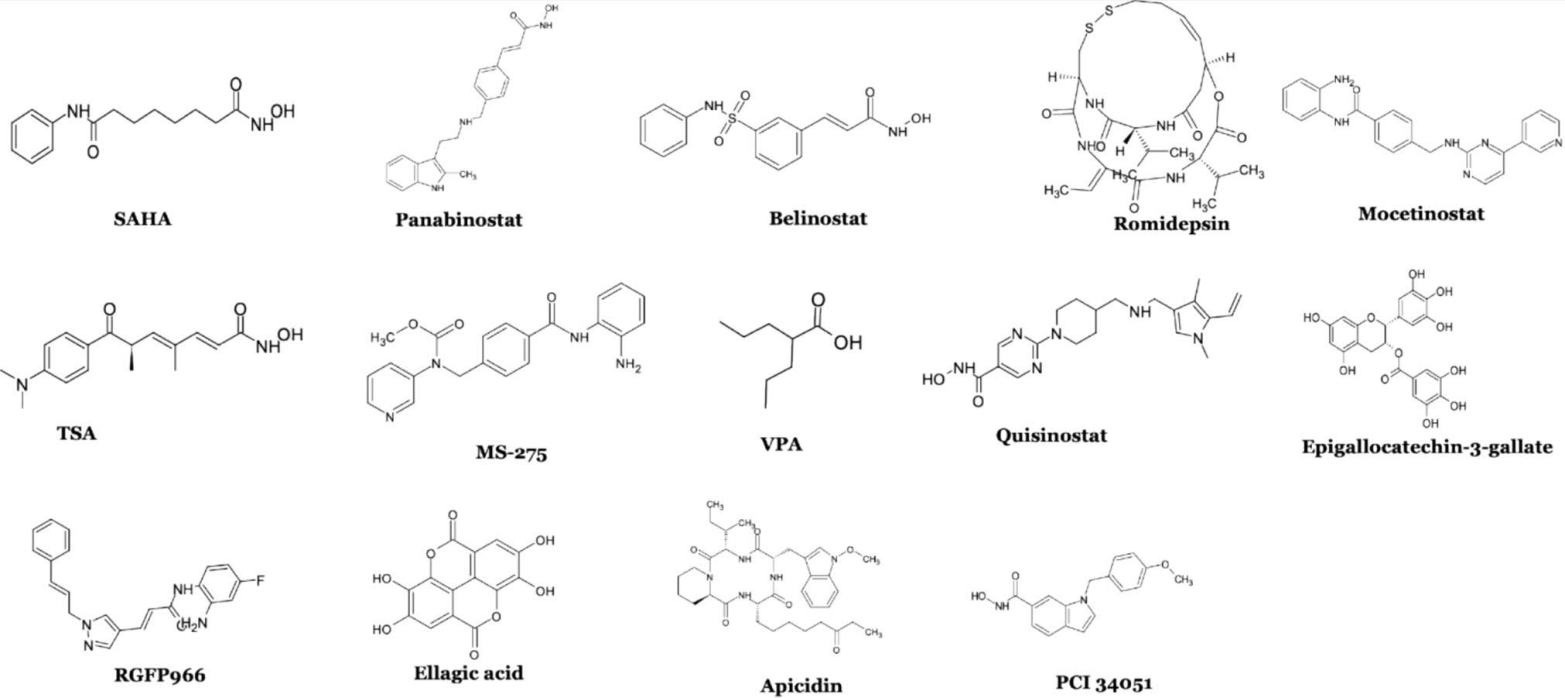
The structures of HDACis used in preclinical cardiovascular disease models

The expression of class I HDAC isoforms, such as HDAC1 and 2, is upregulated in congestive heart failure (CHF) and also in CD90+cardiac fibroblasts. Remarkably, HDAC inhibitors (HDACis) were found to inhibit myofibroblast activation, reverse interstitial cardiac fibrosis in congestive heart failure, and induce cell apoptosis, demonstrating their potential therapeutic value. A decrease in collagen III and α-smooth muscle actin (α-SMA) expression induced by mocetinostat was a marker of a decrease in myofibroblast proliferation. This inhibitor also upregulated two apoptotic genes, *p53* and *p21*, in fibroblasts [13]. These HDACs serve as the catalytic core for coactivator complexes, such as Sin3, nucleosome remodelling deacetylase (NuRD), RE1-silencing transcription factor corepressor (CoREST), and the mitotic deacetylase complex (MiDAC), which increase HDAC1/2 activity [14]. HDAC3 complexes with the silencing mediator of nuclear receptor corepressors. It regulates the redox system, which encompasses mitochondrial metabolism, reactive oxygen species (ROS) sensitivity, and the alteration of antioxidant genes [15]. HDAC8 is a distinctive target for treating CVDs. It plays a distinctive role in the development of cardiac hypertrophy and fibrosis. The HDAC8 inhibitor PCI34051 helps mitigate HDAC8 enzymatic activity [16]. HDACis are well established as anticancer agents and have received FDA approval for the treatment of certain cancers and Duchenne Muscular Dystrophy. Although no HDACis are currently approved for clinical use in cardiovascular diseases, their potential therapeutic effects are investigated in preclinical studies. These studies suggest that HDACis may help regulate key proteins, microRNAs, and signalling pathways involved in inflammation and other pathological processes underlying cardiovascular disorders. In addition, HDACis are valuable tools for examining the role of specific HDAC isoform abnormalities in various cardiovascular conditions. The chemical structures of the most extensively studied HDACis, in preclinical cardiovascular disease models are illustrated in Fig. 2.

This review begins by outlining the molecular mechanisms of HDACs, providing overview on understanding their role in cardiovascular health and disease. It then discusses the major risk factors, diagnostic approaches, and currently available treatments for cardiovascular diseases, emphasizing the importance of HDACs and their inhibitors as emerging therapeutic targets. The review further explores the roles of class I HDAC isoforms in cardiovascular remodelling processes, including their influence on cellular differentiation and pathological changes. The involvement of class I HDAC isoforms in specific cardiovascular disorders such as cardiac hypertrophy, myocardial infarction, atherosclerosis, cardiac fibrosis, myocarditis, vascular calcification, and myocardial oedema is elaborated. The review concludes with a discussion of translational opportunities and future prospects of cardiovascular diseases.

This review focuses on Class I HDACs, which play unique roles in both cardiac remodelling and cardiovascular diseases. These HDACs have been shown to significantly promote the pathogenesis of various cardiovascular conditions, including hypertrophy, heart failure, and MI by modulating the transcriptional activity of key cardiac genes. By understanding the mechanisms through which Class I HDACs function, we can gain insights into potential therapeutic targets for treating CVDs.

## Cardiovascular remodelling and cellular differentiation

Cardiovascular remodelling involves alterations at cellular and molecular levels within the heart, leading to changes in the overall mass and geometry of the heart. This complex process encompasses a range of cellular activities, including myocyte hypertrophy, apoptosis, fibroblast proliferation, and extracellular matrix reorganization [17].

### HDAC1 in cardiovascular remodelling and cellular differentiation

Kruppel-like factor 5 (KLF5), a zinc finger transcription factor, regulates cardiovascular remodelling and is modulated by acetylase p300 and oncogenic regulator SET. HDAC1 negatively regulates KLF5 by directly interacting with it, inhibiting its DNA binding activity and suppressing promoter activation. This interaction prevents KLF5 and p300 from binding to the first zinc finger, highlighting a competitive mechanism. HDAC1-dependent inhibition of KLF5's functions suggests a pivotal role in transcriptional regulation. These findings emphasize HDAC1's influence on KLF5 activity through direct interactions and disruption of p300-mediated modulation [18].

Montgomery and colleagues elucidated the role of HDAC1 and HDAC2 in the development of cardiovascular structures through the proliferation of cardiomyocytes. HDAC2 deletion leads to uncontrolled proliferation of cardiac cells after gastrulation, whereas HDAC1 deletion blocked cardiac morphogenesis and proliferation, by upregulation of *p21*. Deletion of either of these genes does not affect cardiac development directly, but the global deletion of HDAC2 and HDAC1 causes lethal cardiac abnormalities [19]. Cardiomyocyte differentiation is regulated by signalling pathways, including wingless and integrase (WNT) and *Hdac1*. Liu et al. demonstrated that *Hdac1* influences the WNT pathway, using P19CL6 (P19CL6 cells are a subclone of the P19 embryonal carcinoma cells). NK2 homeobox 5 (*Nkx2.5)* is critical for early cardiomyogenesis and is regulated by histone acetylation and methylation. Increased acetylation of histones H3 and H4 accompanies early *Nkx2.5* upregulation, although *Hdac1* downregulation occurs. Overexpressing *Hdac1* reduces *Nkx2.5* and *Gata4* expression, although inhibiting the WNT pathway upregulates *Nkx2.5* and downregulates *Hdac1*. Secreted frizzled-related protein 2 (SFRP2) and glycogen synthase kinase 3 beta (GSK3β) counteract WNT3a and WNT3 to regulate *Hdac1*, highlighting WNT pathway regulates the *Hdac1* in early cardiomyogenesis [20].

Another study revealed that HDAC1 functions as a molecular switch in the tumour necrosis factor α (TNFα) pathway to determine cell survival. An increase in NF-κB transcription was observed in TNFα-treated cells but not in vehicle-treated control cells. HDAC1 is specific for p65, a subunit of NF-κB, which increases the probability of regulating nuclear NF-κB expression via HDAC1. HDAC1-mediated inhibition of NF-κB, which is important for gene transcription, corresponds with shoot-up cell death. This is the first evidence that links HDAC1 and the canonical TNFα pathway for cardiac myocyte survival [21].

Lu and colleagues studied the effect of HDAC1 on mesenchymal stem cells (MSCs). These bone marrow-derived MSCs can be reprogrammed to form cardiomyocytes in the myocardial microenvironment. HDAC1 expression is greater in MSCs than in cardiomyocytes. However, when MSCs were co-cultured, the expression of HDAC1 decreased with time, which promoted the cardiac cell differentiation of MSCs, as indicated by the elevated expression of cardiac troponin T. HDAC is such as suberoylanilide hydroxamic acid (SAHA) and trichostatin A (TSA) promote the differentiation of MSCs into cardiomyocyte-like cells as well as other cell lineages. HDAC1 has a negative effect on the cardiac cell conversion of MSCs into cardiomyocytes. MSCs differentiate into cardiomyocytes in the heart, which might be regulated by HDAC1 [22].

A study demonstrated that the cardiogenic differentiation efficiency of distinct progenitor cell types is increased by inhibitors that target the expression of specific HDAC family members. The effects of HDAC inhibitors on patient cardiac-derived mesenchymal stromal cell (CMC) proliferation, cell lineage specification, cardiovascular differentiation, and cardiogenic program activation were examined with the help of genetic targeting approaches. HDAC1 is essential for promoting cardiogenic transcription, as confirmed by the increased expression of vasculogenic-specific and cardiomyogenic markers. This effect is correlated with and depends on increased acetylation and stabilization of p53. In contrast, CMCs in which HDAC1 was knocked down via short hairpin RNA (shRNA) presented decreased p53 levels. HDAC1 is a potential therapeutic target for enhancing the cardiac reparative capacity of CMCs for cell-based regenerative applications [23]. Moore and colleagues showed HDAC1 affects the secretion of cytokines by CMCs and its impact on endothelial cell function. This study involved transducing CMCs with shRNA constructs to inhibit HDAC1 (shHDAC1) or a control nontargeting construct. The conditioned medium (CM) from these cells revealed that shHDAC1-transduced CMCs secreted more cytokines related to cell growth and differentiation. When this CM was applied to human endothelial cells in vitro, it significantly promoted endothelial cell proliferation and the formation of blood vessel-like structures. The key finding was that basic fibroblast growth factor (bFGF) was upregulated in shHDAC1-transduced CMCs and played a crucial role in these proangiogenic effects [24]. Another study demonstrated that aging in cardiomyocytes downregulated the levels of nuclear receptor corepressor 1 (NCoR1) and HDAC1 and the interactions between them. Toll-like receptor 4 (TLR4), an innate proinflammatory mediator, plays a role in cardiac aging. Ablation of TLR4 attenuated advanced aging and cardiac remodelling by controlling autophagy. TLR4 inhibition increased the levels of NCoR1 and HDAC1. Aging is known to suppress autophagy, as shown by the green fluorescent protein light chain-3B (GFP-LC3B) puncta in neonatal cardiac cells of mice. The inhibitor CLI-095 of TLR4 nullifies these effects. The inhibition of HDAC1 by apicidin prevents the positive response of CLI-095 to aging [25].

Arcidiacono and colleagues studied mouse embryonic stem cell (mESC) differentiation into cardiomyocytes and investigated the effects of histone deacetylase inhibitors on this process, as well as their impact on embryonic hearts. Depletion of HDAC1 in mESCs caused preponed beating of cardiomyocytes and reduced the adherence of embryonic bodies. H4 deacetylation during cardiomyocyte formation in wild-type mESCs decreases in HDAC1-depleted cells following cardiac induction. Furthermore, explanted embryonic hearts respond to HDACi treatment by increasing the acetylation levels of specific histone marks. This suggests that explanted tissue can maintain a hyperacetylation state, which is potentially useful for transplantation strategies, and in transplanted tissue, where acetylation maintains gene upregulation [26]. Another study described a mutant of zebrafish *cardiac truly gone (crg),* an *Hdac1* deficient mutant in which the hearts are smaller due to less proliferation of muscle and ventricular cardiomyocytes in the outflow tract caused by loss of function of the second heart field (SHF). Retinoic acid is a teratogen that epigenetically regulates the expression of *Hdac1* and thus affects the development of the outflow tract (OFT). In embryos deficient in *Hdac1* and cytochrome P450, family 26 (*Cyp26)*, the progenitors of *nkx2.5*^+^ exhibit abnormal expression of the retinoic acid-responsive gene *ripply3*. Upregulation of this gene suppressed vascular cell proliferation and OFT development [27].

Yang and colleagues reported that myocardial troponin 1 (*cTnI*) is an important protein of the heart that is involved in the relaxation and contraction of the heart by regulating the levels of calcium ions in cardiomyocytes. With age, the level of *cTnI* decreases due to increased binding of HDAC1 to the GATA4 promoter region of histone H3. HDAC1 inhibits the acetylation of H3K4, H3K27 and H3K9 at the *cTnI* promoter region. miR-449, an HDAC1 regulator that inhibits *Hdac1* gene expression, leads to increased acetylation of the GATA promoter, thus increasing the expression of *cTnI* in cardiac cells and lowering the risk of cardiac dysfunction [28]. Another study focused on zebrafish, a model organism that can proliferate cardiomyocytes throughout adulthood, but the underlying mechanisms are not known. The mutation induced by N-ethyl-N-nitrosourea (ENU) in the zebrafish mutant baldrian (*balhc050*) caused a reduction in the number of cardiomyocytes. This was due to the change in one base pair in the *Hdac1* gene, that degraded *Hdac1*. Mutation of the gene controlling *Hdac1* expression or *Hdac1* inhibition leads to the underdevelopment of heart muscle in zebrafish embryos and impaired cardiac cell proliferation in adult zebrafish postcardiac injury [29]. Boos and colleagues reported that Hdac1 controls regenerative cardiomyocyte proliferation in both adults and embryos of a zebrafish model. It maintains the normal G1/S and G2/M phase transitions in cardiac muscle cells by regulating the expression of certain regulators involved in the cell cycle, such as p21 and cell division cycle-25 (Cdc25). There is decreased expression of the p21 protein, which normally increases in response to DNA damage, and decreased expression of Cdc25, which results in the downregulation of cyclin-dependent kinase 1 (Cdk1), which causes the G2/M phase transition to be impaired. p21 inhibits the activity of Cdk2, which is responsible for cell cycle progression from G1 to S phase. These effects impact the proliferation of cardiomyocytes [30] (Table 1).

**Table 1:** Genes, proteins, signalling cascades, and the implications of associated HDAC1 in cardiovascular remodelling and survival

Cardiovascular condition	Genes/protein/pathway involved	Inference	References
Cardiovascular remodelling	KLF5, p300	HDAC1 negatively modulates KLF5 activity	[18]
Cardiac morphogenesis	p21	Global deletion of hdac1 and 2 causes lethal cardiac abnormalities	[19]
Cardiomyocyte differentiation	WNT signalling pathway, *Nkx2.5, Gata4,* SFRP2, GSK3β	WNT pathway regulates Hdac1 which is important for cardiomyocyte differentiation	[20]
Cell survival	TNFα pathway, NF-kB pathway	HDAC1 inhibition of NF-kB causes cell death	[21]
Congestive heart failure	α-SMA, collagen III, *p53* and *p21*	HDACi reverses interstitial fibrosis and induces cell apoptosis Decrease in collagen-III and α-SMA expression by Mocetinostat is an indicative of reduced myofibroblast proliferation	[13]
Cardiomyocyte differentiation	cTnT	HDAC1 negatively regulates differentiation of MSCs into CMs SAHA and TSA promotes differentiation of MSCs into various cell lines.	[22]
Cardiomyocyte differentiation	*p53*	HDAC1 is a promising target to strengthen the cardiac reparative potential of CMCs	[23]
CMCs transdifferentiation	bFGF	bFGF is upregulated by HDAC1 silencing and plays a role in their proangiogenic effects	[24]
Cardiac aging	NCoR1, TLR4	HDAC inhibition causes the positive response of TLR4 inhibitor to be cancelled off against aging The inhibition of HDAC1 by apicidin prevents the positive response of CLI-095 to aging	[25]
Cardiomyocyte differentiation		Explanted tissue can maintain a hyperacetylated state, important for transplantation strategies Explanted embryonic hearts respond to TSA/SAHA/VPA treatment by increasing acetylation levels of specific histone marks	[26]
Cardiomyocyte proliferation	*Ripply3, nkx2.5*+	Upregulation of ripply3 suppresses VC proliferation and development of OFT	[27]
Cardiac dysfunction	*cTnI,* GATA4, miR-449	miR-449 inhibits HDAC1 expression and lowers the risk of cardiac dysfunction	[28]
Cardiomyocyte proliferation	*baldrian (balhc050)*	*Hdac1* plays a conserved role in developmental and adult regenerative cardiomyocyte proliferation in the vertebrate heart	[29]
Cardiomyocyte proliferation	p21, cdc25, cdk1, cdk2	p21 inhibits cdk2 activity, which impacts cardiomyocyte proliferation	[30]

In summary the development, remodelling and differentiation of the cardiovascular system, HDAC1 plays a crucial role as an epigenetic regulator. It suppresses KLF5 activity, controls cardiomyocyte proliferation through WNT signalling and cardiac transcription factors like Nkx2.5 and GATA4, and influences survival pathways such as TNFα-NF-κB. In mesenchymal and cardiac stromal cells, HDAC1 inhibits cardiac differentiation and angiogenesis, which can be reversed by HDAC inhibitors. With age, the functioning of HDAC1 decreases, which has a detrimental effect on cardiac function and autophagy; conversely, in models such as zebrafish, inhibiting HDAC1 leads to heart regeneration. With age, HDAC1 also suppresses cardiac genes like cTnI, a process that miR-449 counteracts. These various roles underscore HDAC1's potential as a therapeutic target in cardiovascular disease and regeneration.

### HDAC2 in cardiovascular remodelling and differentiation

Trivedi and colleagues reported that Hdac2 physically interacts with the Gata4 gene with the help of a small homeodomain factor, homeodomain-only protein (Hopx), which stabilizes this interaction. Gata4 activates cell cycle genes, thus promoting the proliferation of cardiomyocytes in mouse embryos. Downregulation of either of these genes causes muscular perforation in mice, whereas homozygous downregulation of both genes leads to muscular ventricular septal defect. Gata4 is directly deacetylated by Hdac2, but downregulation of this acetylation causes hyperacetylation of Gata4, thus promoting cell cycle completion in cardiomyocytes [31]. In the left ventricle (LV) of rats subjected to the two-kidney two-clip method (2K2C), a study revealed increased protein expression of atrial natriuretic factor, HDAC2, HDAC8, and β-myosin heavy chain 4–12 weeks after the operation. This research also examined the aberrant expression of HDACs and evaluated the effects of valproic acid (VPA) on cardiac remodelling. The 2K2C rats treated with VPA presented decreased fibrosis, cardiac hypertrophy and cardiac dysfunction. Moreover, the expression of transforming growth factor-beta 1 (TGF-β1), connective tissue growth factor (CTGF), HDAC2 and HDAC8 is inhibited by VPA. These findings show that both HDAC2 and 8 play important roles in cardiac remodelling and that VPA decreases hypertension, possibly through a decrease in the expression of HDAC2, 8, CTGF and TGF-β1 [32]. Cencioni and colleagues reported that some mESCs express nitric oxide synthase (eNOS), which is required for the endogenous synthesis of nitrogen oxide. This arrangement in some cells of the eNOS/ nitric oxide (NO)-positive subpopulation more efficiently promotes the production of cardiovascular precursors. The mechanism underlying this process involves the activation of mesendodermal genes due to S-nitrosylation of Hdac2, resulting in the inability of Hdac2 to bind to the zinc finger E-box binding homeobox 1 (Zeb1) factor and form the Zeb1–Hdac1 complex. This caused transcriptional repression of the differentiation of mESCs [33]. Milestone and colleagues demonstrated how cryptic transcription produces short RNA sequences in senescent cells. Cryptic transcriptional silencing by Hdac1 and Hdac2 plays a role in the generation of cardiac cells during vertebrate development. Hdac1 and Hdac2 help to switch from mitochondrial oxidative phosphorylation to anaerobic oxidative phosphorylation. Hdac1/Hdac2 loss in developing murine hearts promoted cryptic transcription. Hdac1/Hdac2 work metabolically by deacetylating the H3K23, H3K14 and H3K16 histones, thus silencing cryptic transcription [34].

Neonatal murine cardiomyocytes (NMCMs) treated with anti-*Hdac1* and anti-*Hdac2* siRNAs to predict the effects of HDACs on action potential duration (APD) and the mRNA expression of ion channels show that a slight reduction in the LV ejection fraction increases ventricular effective refractory periods and that prolonged corrected QT interval (QTc) intervals are characteristic of early heart failure. A significant reduction in HDAC2 in LV tissue delays repolarization and may lead to reduced transcript expression of potassium inwardly rectifying channel subfamily J member 2 (*KCNJ2*)/inward rectifier potassium (K_ir_2.1) channels. HDAC2 knockdown reversed AP prolongation in NMCMs. siRNA-mediated downregulation of *Hdac2* reduced potassium voltage-gated channel (*Kcnh2*)/potassium voltage gated channel (K_v_11.1) channel expression. Ion channel expression and ventricular electrical remodelling of the APD are linked to the suppression of HDAC2 in the early stages of heart failure [35] (Table 2).

**Table 2:** Genes, proteins, signalling cascades, and the implications of associated HDAC2 in cardiovascular remodelling and survival

Cardiovascular condition	Genes/protein/pathway involved	Inference	References
Cardiomyocyte proliferation	Hopx, Gata4	Hyperacetylation of the Gata4 promotes the cell cycle completion in cardiomyocytes	[31]
Cardiac remodelling	TGF-β1	HDAC2 and 8 play an important role in cardiac remodelling VPA decreases hypertension, possibly through a decrease in the expression of HDAC2, 8, CTGF and TGF-β1	[32]
mESC differentiation	Zeb1	Interaction between Zeb1, Hdac2, and eNOS is required for early mesendodermal differentiation of naive mESC	[33]
Cardiac development	-	Hdac1 and 2 silence cryptic transcription to promote mitochondrial function during cardiogenesis	[34]
Heart failure	KCNJ2/IK1, Kcnj5/IKACh, or KCNH2/IKr K+channel	Ventricular electrical remodelling of APD and ion channel expression in early stages of heart failure are linked to suppression of HDAC2	[35]

In summary cardiac development and remodelling, HDAC2 is essential. Through its interaction with GATA4 and Hopx, it regulates the proliferation of cardiomyocytes, and its absence results in ventricular defects. In hypertensive hearts, HDAC2 shows increased regulation, and its inhibition through valproic acid leads to a reduction in fibrosis and dysfunction. In stem cells, S-nitrosylation of HDAC2 enhances cardiovascular differentiation by interrupting its association with Zeb1. In developing hearts, HDAC2 also inhibits cryptic transcription and aids metabolic transitions. Its downregulation changes the expression of ion channels and prolongs action potentials, playing a role in early heart failure and highlighting its importance for cardiac structure and function.

### HDAC3 in cardiovascular remodelling and differentiation

Deletion of HDAC1 may cause proliferation defects, leading to embryonic lethality. *Hdac3* is part of a different corepressor complex that allows it to play a distinct role in myocyte proliferation and cardiac development. *Hdac3*-Tg mice exhibit elevated *Hdac3* mRNA expression and a subsequent increase in HDAC activity. Acetylated H4 levels were reduced in these mice. These elevated levels of *Hdac3* impact the expression of Hdac1 and Hdac2. The lumens in the left and right ventricles are completely lost in these mice, and elevated nucleation is observed in the myocytes. *Hdac3* specifically regulates cyclin-dependent kinase inhibitors. Unlike *Hdac2-Tg* mice, *Hdac3-Tg* mice do not exhibit cardiac hypertrophy, which indicates that *Hdac3* is a unique regulator of cardiac cell proliferation [36]. HDACs cause perinatal lethality from dilated cardiomyopathy and cardiac arrhythmia, along with increased cardiac and skeletal muscle gene expression. Cardiac-specific Hdac3 deletion leads to hypertrophy, fibrosis, mitochondrial dysfunction, and decreased cardiac performance. Cardiomyocyte-restricted hdac3 deletion (*Hdac3cko*) mice presented an increased heart weight/body weight ratio, disorganized myofibrils, diminished contractility, and elevated cardiac stress markers. *Hdac3* independently regulates cardiac growth and metabolism, affecting glucose transporter type 1 and 4 (*GLUT1* and *GLUT4)* expression and peroxisome proliferator-activated receptor alpha (PPARα) responsive genes. *Hdac3cko* mice exhibit increased myocardial triglycerides, elevated free radicals, and reduced nicotinamide adenine dinucleotide (NADH) oxidase activity, indicating that Hdac3 is crucial in myocardial energy metabolism [37]. Cavasin and colleagues explored how class I HDACs, specifically HDAC1, HDAC2, and HDAC3, influence cardiopulmonary and vascular remodelling. Their study on inhibitors like MGCD0103 and MS-275 showed that MGCD0103 enhances pulmonary artery acceleration time, reduces systolic notching in the pulmonary artery flow envelope, decreases smooth muscle cell proliferation, and lowers right ventricular hypertrophy. Additionally, this inhibitor prevents cell death in the right ventricle, aiding in maintaining contractile function [10].*Hdac3* is responsible for the regulation of mitochondrial functions and lipid metabolism in the adult heart. *Hdac3* regulates the T-box family gene *Tbx5*, which can cause Holt–Oram syndrome, resulting in atrial and ventricular septal defects. *Hdac3*^*Nkx2−5KO*^ embryos are generated by deleting *Hdac3* in cardiac progenitor cells. These patients exhibit various cardiac deformities, such as membranous ventricular septal defects and hypoplastic ventricular walls. *Hdac3* is present at 13 sites in E8.5 wild-type hearts, 11 of which are occupied by both Tbx5 and Hdac3. Hdac3 deletion causes the upregulation of *Myh7, Tnni2,* and *Tnnt2* genes*,* which is hindered by *Tbx5* knockdown. These findings suggest that *Hdac3* represses the transcription of *Tbx5* during early cardiac cell proliferation. Cotransfection of EP300 has been shown to increase Tbx5 acetylation, whereas cotransfection with HDAC3 reduces acetylation. *Hdac3* regulates *Tbx5* function in cardiogenesis [38]. HDAC3 is crucial for the development of structures from the secondary heart field, influencing extracellular matrix (ECM) stability and endothelial-to-mesenchymal differentiation. HDAC3 silences TGF-β1 via PRC2 recruitment and H3K27 trimethylation. *Hdac3*^*Isl1KO*^ embryos exhibit fragmented elastic fibers and abnormal semilunar valves, although atrioventricular valves are unaffected. *Hdac3*^*Mef2CKO*^ mice show semilunar valve defects with uneven proteoglycan expression. HDAC3-null embryos have increased SMAD2/3 phosphorylation, enhancing TGF-β signalling. Overall, HDAC3 regulates TGF-β in smooth muscle cells to prevent congenital heart defects [39].

Blakeslee and colleagues reported that when cultured cardiomyocytes are treated with HDAC inhibitors, c-Jun amino–terminal kinase-interacting protein-1 (JIP1) mRNA and protein expression are induced. Kinesin family member 5A (KIF5A), a member of the kinesin heavy chain family, is regulated by JIP1, which is induced by an HDAC inhibitor. Evidence of a regulatory circuit that is independent of HDACs has shown that this circuit promotes the formation of JIP1:KIF5A microtubrotubule complexes. These findings indicate that microtubular transport in the heart can be regulated by HDAC inhibitors [40]. Another group studied the importance of *Hdac3* in developing endocardial cells. Knocking out *Hdac3* in the endocardial cells of mice results in early death of the embryo via a hypotrabeculation phenotype. The expression of several extracellular matrix components, such as collagens, is reduced in *Hdac3* knockout (KO) endocardial cells. Trabecular myocardial growth in endocardial cells is promoted by HDAC3, which triggers the *TGF-ß* signalling pathway via the repression of miR-129-5p. miR-129-5p is highly upregulated in *Hdac3* KO mouse cardiac endothelial cells and hearts [41] (Table 3).

**Table 3:** Genes, proteins, signalling cascades, and the implications of associated HDAC3 in cardiovascular remodelling and survival

Cardiovascular condition	Genes/protein/pathway involved	Inference	References
Cardiomyocyte proliferation	Cdkn1a, Cdkn1b, Cdkn1c, Cdkn2b, and Cdkn2c	HDAC3 acts as a novel regulator of cardiomyocyte proliferation during cardiac development	[36]
Cardiac development	GLUT1, GLUT4, PPARα	Hdac3 plays a crucial role in myocardial energy metabolism	[37]
Cardiopulmonary and vascular remodelling	-	HDACi blocks cell death of the right ventricle and inflammation and help in maintaining the contractile function of these cells MGCD0103 blocks cell death in the right ventricle and inflammation and helps maintain the contractile function of these cells. Similar results are shown by MS-275	[10]
Cardiogenesis	*Myh7, Tnni2* and *Tnnt2, Tbx5*	Hdac3 plays a role in regulating Tbx5 function in cardiogenesis	[38]
Cardiac development	PRC2, EZH2, EED and SUZ12, TGF-β1 pathway	Epigenetic dysregulation within the second heart field is a predisposing factor for congenital heart disease	[39]
Cardiac development	JIP1, KIF5A	Microtubular transport in the heart can be regulated by HDAC inhibitors	[40]
Endocardial development	miR-129-5p, *TGFβ* pathway	Endocardial HDAC3 promotes trabecular myocardium growth by stimulating the TGFß signalling pathway	[41]

In summary myocyte proliferation, metabolism, and gene expression are regulated by HDAC3, which is essential for heart development and remodelling. Its removal leads to cardiac defects, fibrosis, metabolic dysfunction, and valve abnormalities due to disrupted TGF-β and ECM signalling. HDAC3 represses Tbx5 and has a role in modulating microtubule transport. It is essential for the preservation of cardiac structure and function. The study highlights that HDAC8 remains relatively underexplored in the context of cardiovascular remodelling and cellular differentiation. Further research is essential to understand its mechanisms and therapeutic potential. Expanding investigations on HDAC8 may reveal novel insights for cardiovascular disease treatment.

## HDAC isoforms associated with cardiovascular disorders

### Cardiac hypertrophy

Cardiomyopathy refers to diseases affecting the heart muscle, impairing its ability to pump blood and potentially leading to arrhythmias, heart failure, and serious cardiovascular issues. In dilated cardiomyopathy, the heart muscle weakens and the heart enlarges, which reduces the efficiency of blood pumping and can cause leaky heart valves. Cardiac hypertrophy occurs when the heart muscle, particularly the myocardium, thickens due to increased workload or stress on the heart [42]. Restrictive cardiomyopathy is a rare condition in which the ventricles become stiff, preventing proper blood flow and leading to high pressure in the ventricles. It is the least common form of cardiomyopathy observed in clinical settings. Restrictive cardiomyopathy can affect one or both ventricles. Nonetheless, elevated atrial pressure frequently results in atrial hypertrophy [43].

#### HDAC1

Zhao and colleagues described restrictive cardiomyopathy as a condition where normal heart tissue is replaced by scar tissue, causing ventricular dysfunction and atrial enlargement. Myosin filaments bind to cardiac troponin I, essential for muscle contraction. The mutant cardiac troponin I (cTnIR193H) interacts with HDAC1, reducing PDE4D levels in cardiomyocytes through promoter degradation, which contributes to the development of restrictive cardiomyopathy [44]. The Yi–Xin–Shu (YXS) capsule, a drug suggested by traditional Chinese medicine (TCM), is commonly applied in the treatment of cardiac hypertrophy by regulating the expression of GATA4, HDAC1, and retinoblastoma (RB) and the corresponding signalling pathway. YXS inhibits the abnormally high expression of GATA4 in cardiovascular hypertrophy, which can be regulated by interactions between HDAC1 and the general control nonrepressed 5 protein (GCN5). YXS suppresses the abnormal elevation of HDAC1 in cardiovascular hypertrophy. YXS increases the expression of RB, whose expression is decreased in cardiac hypertrophy [45].

Li and colleagues studied epigallocatechin-3-gallate (EGCG), an inhibitor of HDAC1, which works by decreasing the binding of HDAC1 and increasing the binding of acH3K9 or acH3K14 to the promoter regions of nuclear respiratory factor 1 (NRF1) and peroxisome proliferator-activated receptor-gamma coactivator (PGC-1α). HDAC1 inhibition, upregulated the NRF1, mitochondrial transcription factor 1 (TFAM), and FUN 14 domain containing 1 (FUNDC1). NRF1 histone acetylation maintains the mitochondrial homeostasis associated with cardiac hypertrophy [46].

#### HDAC2

Heat shock proteins (HSPs) are proteins produced in response to stressful conditions. The induction of hypertrophy in mice by heat shock activated HSP70, which is physically associated with class I HDACs and HDAC2, and increased its expression, thus providing a signal for hypertrophy. Mutant mice lacking HSP70 show blunted HDAC2 activation and a hypertrophic response. The hypertrophic phenotype is blocked by siHDAC2 or negative control HSP70 [47]. Eom and colleagues examined HDAC2's role in cardiac hypertrophy using transgenic mice. Mice with high HDAC2 levels displayed hypertrophy, although those with the phosphorylation-resistant HDAC2 S394A mutation did not. Hypertrophic cardiomyopathy mice had increased HDAC2 S394 phosphorylation. CK2α1 inhibitors blocked this phosphorylation and activity. Treating CK2α1-transgenic mice with TSA reduced hypertrophy. HDAC and CK2 inhibitors, along with HDAC2 S394A, lowered CK2α1 expression, which promotes cardiomyocyte hypertrophy [48]. The interactions between proteins associated with hypertrophic gene regulation, including mSin3A, HDACs 1 and 2, O-linked β-N-acetylglucosamine transferase (OGT), and repressor element-1 silencing transcription factor (REST), as well as the role of O-linked β-N-acetylglucosamine (O-GlcNAc), a subform of glucose that increases glucose levels in cells, were studied. Diabetic hearts exhibit increased levels of O-GlcNAc, which is further increased by exercise. Diabetic hearts exhibit increased HDAC2 mRNA expression. However, they presented decreased protein levels of mSin3A, HDAC1, and HDAC2. In sedentary diabetic hearts, there is a reduced association between mSin3A and HDAC1 and between OGT and HDAC2 when compared with nondiabetic controls, which is normalized by exercise [49].

Another study revealed the importance of interclass connections in the progression of cardiac hypertrophy. A decrease in the phosphorylation of S394 is shown by acetylation-resistant HDAC2 K75R. In Hdac5-null mice, Hdac5 directly deacetylases Hdac2. The introduction of a mutant of Hdac2 in cardiomyocytes reduces the antihypertrophic effect caused by the upregulation of HDAC5. In hypertrophic hearts, HDAC2 acetylation is regulated by two proteins, HDAC5 and p300/CREB-binding protein (CBP)-associated factors [50].

Ying and colleagues examined how Janus kinase 2 (Jak2) contributes to cardiac hypertrophy from pressure overload. They found that Ang-II re-expresses foetal genes atrial natriuretic peptide (ANP) and brain natriuretic peptide (BNP)in cardiomyocytes, an effect blocked by Jak2 inhibitor AG-490 and HDAC2 inhibition. Pressure overload and Ang-II also cause HDAC2's nuclear export, which is inhibited by AG-490, indicating Jak2's role in the hypertrophic response [51]. Eom and colleagues noted that HDAC2 acts as a hypertrophic mediator, although HDAC4, 5, and 9 serve as negative regulators. Phosphorylation-induced redistribution of HDACs is essential for their function, with HDAC2 becoming activated during cardiac hypertrophy progression [52].

Another study used intraperitoneal isoproterenol (ISO) injection to establish a rat hypertrophy model. When the rats were treated with palmatine, the transcription of *BNP, HDAC2 and ANP* decreased, but that of *HDAC5* decreased, and ISO-induced hypertrophy decreased in the rats. The expression of Krüppel-like factor 4 (*KLF4)* and inositol polyphosphate-5-phosphatase F (*INPP5F)* which are downstream effector genes, is restored upon treatment with palmatine [53]. Yan and colleagues investigated whether sphingosine-1-phosphate (S1P) downregulates the activity of HDAC2 and could ameliorate cardiac function in mice with transverse aortic constriction (TAC) and investigated the underlying mechanism involved. KLF4 upregulates S1P, and S1P decreases the activity of only HDAC2 but does not affect its expression and increases the acetylation of histone H3. These suppressive effects of S1P are independent of S1P receptor 2 (S1PR2), but S1PR2 might be involved in its antihypertrophic effects. These findings indicate that heart diseases caused by cardiac hypertrophy can be treated with S1P [54].

Yoon and colleagues identified functional complex proteins of HDAC2 using peptide pull-down and immunoprecipitation assays. Serine/threonine-protein phosphatase 2A (PPP2CA), the catalytic subunit of protein phosphatase 2 (PP2A) and a binding partner of HDAC2, prevents HDAC2 phosphorylation. Although PP2A inhibitors lead to hypertrophy, overexpression of PPP2CA downregulates this response. Phosphorylation of HDAC2 S394 is crucial for the antihypertrophic response. In PPP2CA transgenic mice, cardiac hypertrophy and fibrosis from isoproterenol are minimal, but HDAC2 S394E expression induces hypertrophy. Thus, PP2A is a key regulator of HDAC2 activity [55]. Another study shows that knocking down HSP70 reduces HDAC2 S394 and S422/424 but forces the activation of HSP70 to increase HDAC2 S394 phosphorylation but not S422/424 phosphorylation. Casein kinase 2a1 increases HDAC2 binding to Hsp70 by phosphorylating HDAC2 S394, whereas dephosphorylation of PP2CA by the catalytic subunit has the opposite effect. Dephosphorylation of HDAC2 is prevented by HSP70, which decreases the binding of HDAC2 to PP2CA. The binding of HSP70 to HDAC2 is disrupted by 2-phenylethynesulfonamide (PES) phosphorylation, and the activation of HDAC2 S394 is reduced [56].

Ang II-induced hypertrophy in cardiomyocytes can be blocked by the overexpression of the microtubule-associated monooxygenase calponin and LIM domain containing 3 (MICAL-3) protein, which reduces HDAC2 activity although prominently increasing the level of ROS in cardiomyocytes. At the S394 site, the phosphorylation of HDAC2 is reduced by the interaction between HDAC2 and CK2α1, which is affected when MICAL3 competes with HDAC2. The activity of HDAC2 is inhibited by MICAL3 through self-generated ROS; therefore, in myocardial hypertrophy, the positive regulation of HDAC2 is inhibited, and the hypertrophy of myocardial cells is inhibited [57].

#### HDAC8

In mice infused with angiotensin (Ang II) and subjected to transverse aortic constriction (TAC), a decrease in miR-21-3p expression is seen. Ang-II- and TAC-induced cardiac hypertrophy reduces miR-21-3p expression. Recombinant adeno-associated virus (rAAV)-miR-21-3p was administered through the veins of mice to observe the importance of miR-21-3p in cardiac hypertrophy. The hypertrophy caused by TAC and Ang II is decreased when miR-21-3p is overexpressed, as detected via biomarker and cardiac function measurements. Western blot analysis revealed that miR-21-3p silenced HDAC8. When HDAC8 is re-expressed, miR-21-3p decreases, and the upregulation of p-Gsk3β and p-Akt expression moderately represses cardiac hypertrophy conditions. The expression of miR-21-3p is a therapeutic target [58].

Zhao and colleagues demonstrated that HDAC8 overexpression induces cardiac hypertrophy, which the HDAC8 inhibitor PCI34051 can reduce. Isoproterenol treatment in mice increased cardiac hypertrophic and fibrosis-related gene expression, activating the p38 MAPK pathway due to HDAC8 overexpression. Selective HDAC8 inhibition or knockdown suppressed these effects, while p38 MAPK and SB203058 inhibitors decreased BNP and ANP levels regulated by HDAC8 [16]. HDAC8 inhibition by the inhibitor PCI34051 alleviates cardiac hypertrophy or hypertension induced by isoproterenol and Ang-II. HDAC8 knockout mice show reduced inflammation, as evidenced by Rela downregulation and NFKB inhibitor alpha (nfkbia) upregulation in cardiomyocytes. HDAC8 expression was directly related to the expression levels of angiotensin-converting enzyme 1 (*Ace1*) and angiotensin II receptor type 1 (*Agtr1*), whereas the upregulation of HDAC8 downregulated *Ace2* and *Agtr2* in post-TAC mice. The renin-angiotensin‒aldosterone system *(RAAS)* is reportedly activated in patients with heart failure. This process is promoted by HDAC8 [59].

#### Shared mechanistic roles of multiple HDAC isoforms in cardiac hypertrophy

Autophagy is a major component of cardiac remodelling in response to cardiac hypertrophy. Autophagy is the natural response to hypertrophy, but excess autophagy caused by Beclin 1 needs to be controlled by HDAC2 inhibitors. Inhibitors such as TSA help reduce the load on the heart and help reduce the activated autophagic response. Studies have also revealed that HDAC inhibitors are useful for restoring the normal function of cardiomyocytes in the hypertrophic hearts of mice [60]. Fibroblast proliferation causes aberrant thickening of heart valves, leading to cardiac fibrosis. Williams and colleagues reported that the inhibition of HDAC class I has the potential to suppress cardiac fibrosis induced by Ang-II. It affects two main cell types: cardiac fibroblasts and bone marrow-derived fibrocytes. These inhibitors reduce the cardiac fibroblast population by suppressing the p15 and p57 genes, which encode CDK inhibitors. They also block the expression of these proteins; thus, inhibiting the proliferation of fibroblasts. Inflammation is suppressed by HDAC inhibitors, which are known causes of fibrosis [61].

Kee and colleagues found that HDAC inhibitors, including sodium valproate, can prevent hypertrophy. In nephrectomized rats with implanted 4-Deoxycorticosterone acetate (DOCA) strips, valproate reduced cardiac hypertrophy and fibrosis, measured by the heart weight/body weight (HW/BW) ratio. It also decreased the activity of HDAC8 and HDAC6. Thus, selective inhibitors of these HDACs may help to treat cardiac hypertrophy [62]. Cancer cell growth can be restricted by modifying transcriptional responses by class I HDACs. Through genetic and pharmacological methods, it has been shown that inhibiting HDACs, particularly class I HDACs, also inhibits mTOR activity, which reduces pathological cardiac hypertrophy. Moreover, tuberous sclerosis complex 2 (TSC2), an mTOR inhibitor, increases when class I HDACs are inhibited through transcriptional mechanisms in cardiomyocytes. These findings reveal a way to control mTOR activity and aid in the development of HDACis for CVD treatment because of their translational activities [63].

**Fig. 3:**
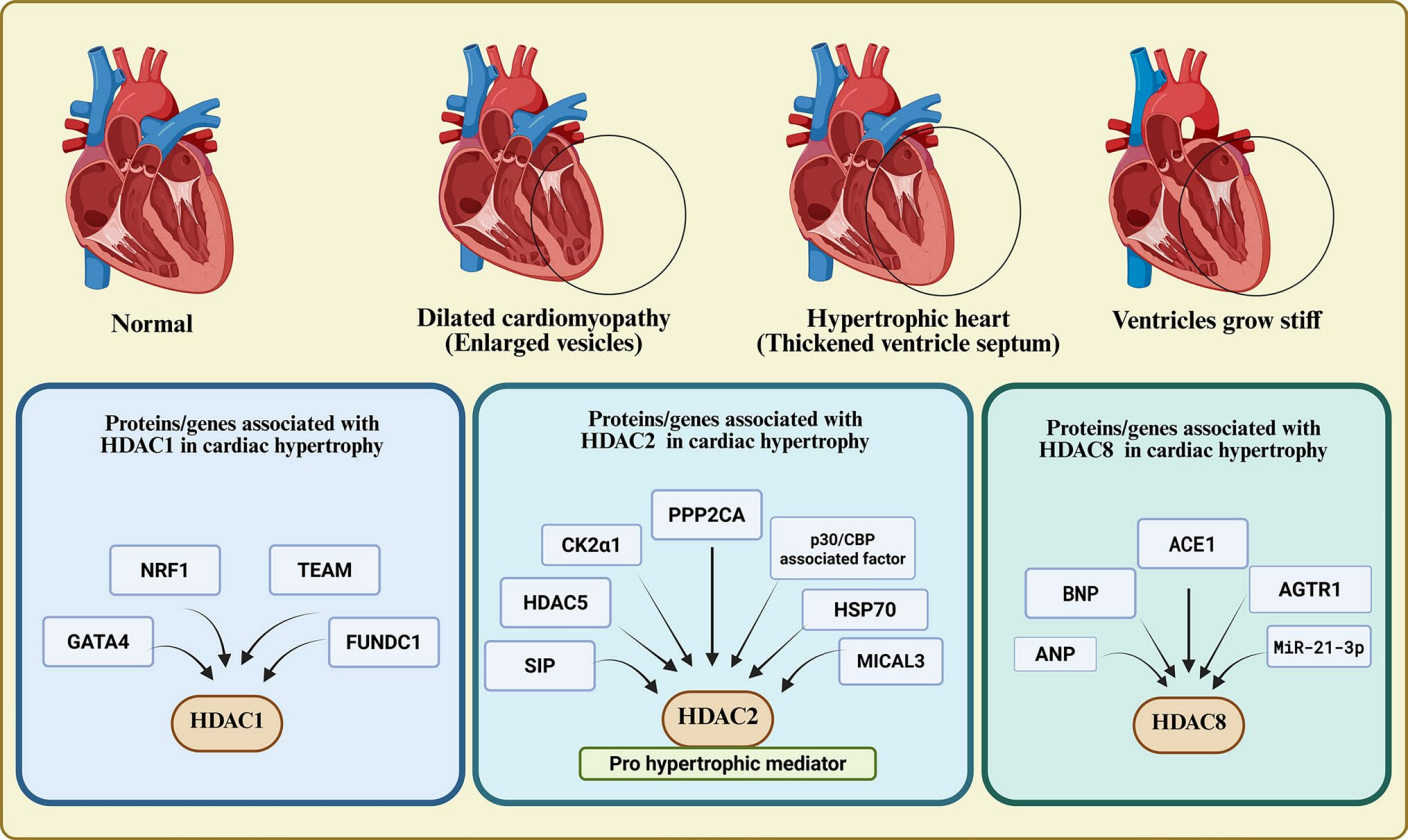
HDAC association with proteins and genes regulates cardiac hypertrophy

Hypertrophic induction pathways are regulated by class I and II HDACs. The HDAC inhibitor magnesium valproate (MgV) primarily targets class I HDACs, effectively inhibiting hypertrophic HDAC expression, as confirmed by mRNA studies. Selective class I HDAC inhibitors can manage hypertrophy, while dual inhibitors offer broader regulatory options [64]. Zhang and colleagues reported that the Ca^2+^/calmodulin-dependent kinase II (CaMKII) pathway plays a major role in leading to heart failure. CAMKII inhibits class IIa HDACs, such as HDAC4 and HDAC5, which are known to prevent cardiac hypertrophy by suppressing the expression of myocyte enhancer factor-2 (MEF2), a prohypertrophic transcription factor. HDAC class I is known as a promoter of cardiac hypertrophy, and its incubation with CaMKIIδ, Ca^2+^/calmodulin and ATP increases HDAC class I activity. It works *via* the phosphorylation of HDAC1, HDAC2 and HDAC3, whereas the phosphorylation of HDAC8 causes its inhibition. Quisinostat, an HDAC1 inhibitor, show promise in relieving CAMKII-induced cardiac hypertrophy. In addition to this inhibitor, other general class I inhibitors, such as apicidin, also ameliorate the effects of myocardial hypertrophy (Table 4; Fig. 3) [65].

**Table 4:** Genes, proteins, signalling cascades, and the implications of associated HDACs in cardiac hypertrophy

Isoform	Protein/gene related to isoform	Histological and pathological inference	References
HDAC1	cTnI, PDE4D	PDE4D expression reduction causes restrictive cardiomyopathy	[44]
	GATA4, GCN5	YXS increases the expression of RB whose expression is decreased in cardiac hypertrophy	[45]
	PGC-1α, NRF1, TFAM, FUNDC1	NRF1 histone acetylation maintains mitochondrial homeostasis associated with cardiac hypertrophy EGCG works by decreasing the binding of HDAC1 and increasing the binding of acH3K9 or acH3K14 to the promoter regions of NRF1 and PGC-1α	[46]
HDAC2	HSP70	The hypertrophic phenotype is blocked by siHDAC2 or negative Hsp70	[47]
	CK2α1	Chemical inhibitors of both HDAC and CK2 as well as HDAC2 S394A blunted the overexpression of CK2α1 which caused hypertrophy in cardiomyocytes Treatment of CK2α1-transgenic mice with TSA, attenuates hypertrophy	[48]
	mSin3A, REST, OGT, O-GlcNAc	An increase in total cardiac O-GlcNAc is a mechanism by which exercise benefits type 2 diabetic hearts	[49]
	HDAC5 and p300/CBP	The acetylation of HDAC2 is regulated by p300/CBP-associated factor and HDAC5 during the progression of cardiac hypertrophy	[50]
	Ang-II, Jak2, BNP	Jak2 has HDAC2 as downstream effector to mediate hypertrophic response by Ang-II or pressure overload TSA prevents Ang-II mediated re-expression of foetal genes	[51]
	-	HDAC2 activates during development of cardiac hypertrophy	[52]
	*BNP,KLF4,ANP, INPP5F,HDAC2, HDAC5*	Palmatine possesses promising therapeutic potential against hypertrophy	[53]
	S1P, KLF4, SIPR1, SIPR2	Heart diseases caused by cardiac hypertrophy can be treated with S1P	[54]
	PP2A and PP2CA	PP2A is a major regulator of HDAC2 activity	[55]
	HSP70, PES, PP2CA	Inhibition of HSP70 can be used to treat cardiac hypertrophy	[56]
	CK2α	In myocardial hypertrophy positive regulation of HDAC2 is inhibited and inhibiting the hypertrophy of myocardial cells	[57]
HDAC8	miR-21-3p, Ang II, Gsk3β, Akt	Modulating miR-21-3p levels can be used in treating cardiac hypertrophy	[58]
	P38, MAPK pathway, ANP, and BNP	HDAC8 is a promising target for treating cardiac hypertrophy and fibrosis by regulating the p38 MAPK pathway PCI30451 suppresses cardiac hypertrophy and fibrosis	[16]
	NF-kBIα, ACE1, AGTR1	HDAC8 is a potential novel therapeutic target for heart failure accompanied by pathological lung diseases HDAC8 inhibition by the inhibitor PCI34051 alleviates cardiac hypertrophy or hypertension induced by isoproterenol and Ang-II	[59]
-	Beclin 1	HDACi help to bring back the normal function of cardiomyocytes in hypertrophic hearts TSA helps reduce the load on the heart and help reduce the activated autophagic response	[60]
Class I HDACs	Ang-II, p15 and p57	Inflammation is suppressed by HDACi	[61]
Class I and II HDACs	-	Selective HDAC8 and 6 inhibitors can be used to treat cardiac hypetrophy TSA, sodium valproate, and butyrate can prevent hypertrophy	[62]
Class I HDACs	mTOR, TSC2	TSC2 levels increase when class I HDACs are inhibited	[63]
Class I and II HDACs	-	Cardiac hypertrophy can be controlled by selective class I HDAC inhibitor Improvement in hypertrophy is due to HDAC inhibition by MgV	[64]
Class I HDACs	CAMKII pathway	Quisinostat, an HDAC1 inhibitor showed promise in relieving the effects of CAMKII-induced cardiac hypertrophy apicidin, also ameliorate the effects of myocardial hypertrophy	[65]

In summary, HDACs, especially those from class I such as HDAC1, HDAC2, and HDAC8, are key players in the promotion of cardiac hypertrophy through the activation of prohypertrophic signalling pathways and the repression of protective genes. Although HDAC2 is activated by stress signals (such as HSP70 and CK2α1) and promotes hypertrophic gene expression, HDAC1 is associated with restrictive cardiomyopathy and mitochondrial dysfunction. HDAC8 plays a role in fibrosis and hypertrophy through the RAAS and MAPK pathways. On the other hand, class IIa HDACs (such as HDAC4 and HDAC5) inhibit hypertrophy. The inhibition of HDACs using substances such as EGCG, sodium valproate, PCI34051, or traditional Chinese medicine has demonstrated potential in decreasing cardiac hypertrophy, fibrosis, and inflammation, suggesting possible therapeutic approaches for heart disease.

### Myocardial infarction

Myocardial ischemia has functional, electrical, metabolic, and structural consequences owing to a lack of coronary blood flow. MI refers to the death of myocardial tissue due to ischemia and a lack of delivery of oxygen to the myocardial tissue [66].

#### HDAC1

Herr and a group focused on selectively inhibiting class I HDACs via MS-275 during reperfusion to improve heart recovery. In rat hearts that underwent ex vivo I/R injury with or without Entinostat (MS-275) treatment during reperfusion, MS-275 greatly reduced I/R injury, improving left ventricular (LV) function and tissue viability by the end of reperfusion. Surprisingly, researchers have reported that HDAC1 is present in cardiomyocyte mitochondria but is absent in fibroblasts or endothelial cells. Selective inhibition of mitochondrial HDAC1 provides the same level of protection as MS-275 does, whereas an inhibitor excluding mitochondrial HDAC1 does not. The effects of mitochondria-excluded and mitochondria-restricted inhibitors are compared with those of MS-275. These effects are related to a decrease in succinate dehydrogenase complex flavoprotein subunit A (SDHA) activity and a subsequent reduction in metabolic ROS production during reperfusion [67].

Nong and colleagues studied how HDAC1 plays a role in myocardial injury in septic mice. HDAC1 levels are increased in the myocardial tissues of mice with sepsis, and these proteins bind to miR-124-5p, which leads to its downregulation. Reestablishment of miR-124-5p improves the function of the cardiac system, reduces cardiomyocyte apoptosis, and attenuates myocardial injury. The levels of high-mobility group box chromosomal protein 1 (HMGB1), which are inhibited by increased levels of miR-124-5p and the downregulation of HDAC1, are dramatically increased in the myocardium in severe sepsis [68]. Another study demonstrated that the expression of the peptidase inhibitor 16 (PI16) protein is increased in heart diseases such as myocardial infarction and heart failure. PI16 overexpression prevents cardiomyocyte apoptosis and cardiac remodelling by downregulating the Wnt3a/β-catenin pathway and inhibiting HDAC1 expression, whereas knockdown of PI16 leads to an increase in cardiomyocyte apoptosis. PI16 is responsible for inhibiting cardiac fibroblast fibrosis by reducing the expression of HDAC1. HDAC1 is an important transcriptional regulator of the Wnt3a/β-catenin pathway, and its inhibition has a beneficial effect on myocardial injury. By entering the nucleus and interacting with some nuclear factors, PI16 may regulate HDAC1 levels or interact with mRNAs to promote HDAC1 degradation. HDAC1 inhibition by PI16 improves LV remodelling and heart function after MI [69].

#### HDAC3

Mani and colleagues, following MI, observed LV dilation and pump dysfunction as part of the left ventricular remodelling process. Damage to the LV is caused by post-MI induction of matrix metalloproteinases (MMPs), mainly MMP-2 and MMP-9. Pump dysfunction, LV dilation and activation of the MMP-9 gene promoter are decreased in mice treated with HDAC inhibitors of class I/IIb, such as SAHA or TSA, when compared with those in nontreated mice. The activity of class I HDAC isoforms is strongly increased post-MI. Post-MI levels of MMP-2 and MMP-9 are reduced upon treatment with PD-106 (a class I inhibitor), and LV pump dysfunction and dilation are reduced post-MI [70]. Su and colleagues reported that H_2_O_2_ exposure decreases cardiomyocyte viability and miR-132-3p levels while inducing apoptosis, elevating Bax, cleaved caspase 3, and ROS levels. The miR-132-3p mimic protects cardiomyocytes by activating oxidation-related genes like B cell lymphoma-extralarge (Bcl-xL), peroxiredoxin-2 (Prdx2), and Hsp70 via HDAC3 inhibition and H3 acetylation. Hypoxia induces taurine upregulated gene-1 (TUG1), which reduces cell viability, increases apoptosis and ROS, and suppresses Bcl-xL and Prdx2 expression. Silencing TUG1 enhances Hsp70 expression, reduces HDAC3 levels, and promotes H3K9 acetylation. TUG1 competes with miR-132-3p for binding to HDAC3, and ischemia upregulates TUG1 while downregulating miR-132-3p. The HDAC3 inhibitor RGFP966 mitigates H_2_O_2_-induced damage by promoting histone acetylation and reducing ROS [71].

**Fig. 4:**
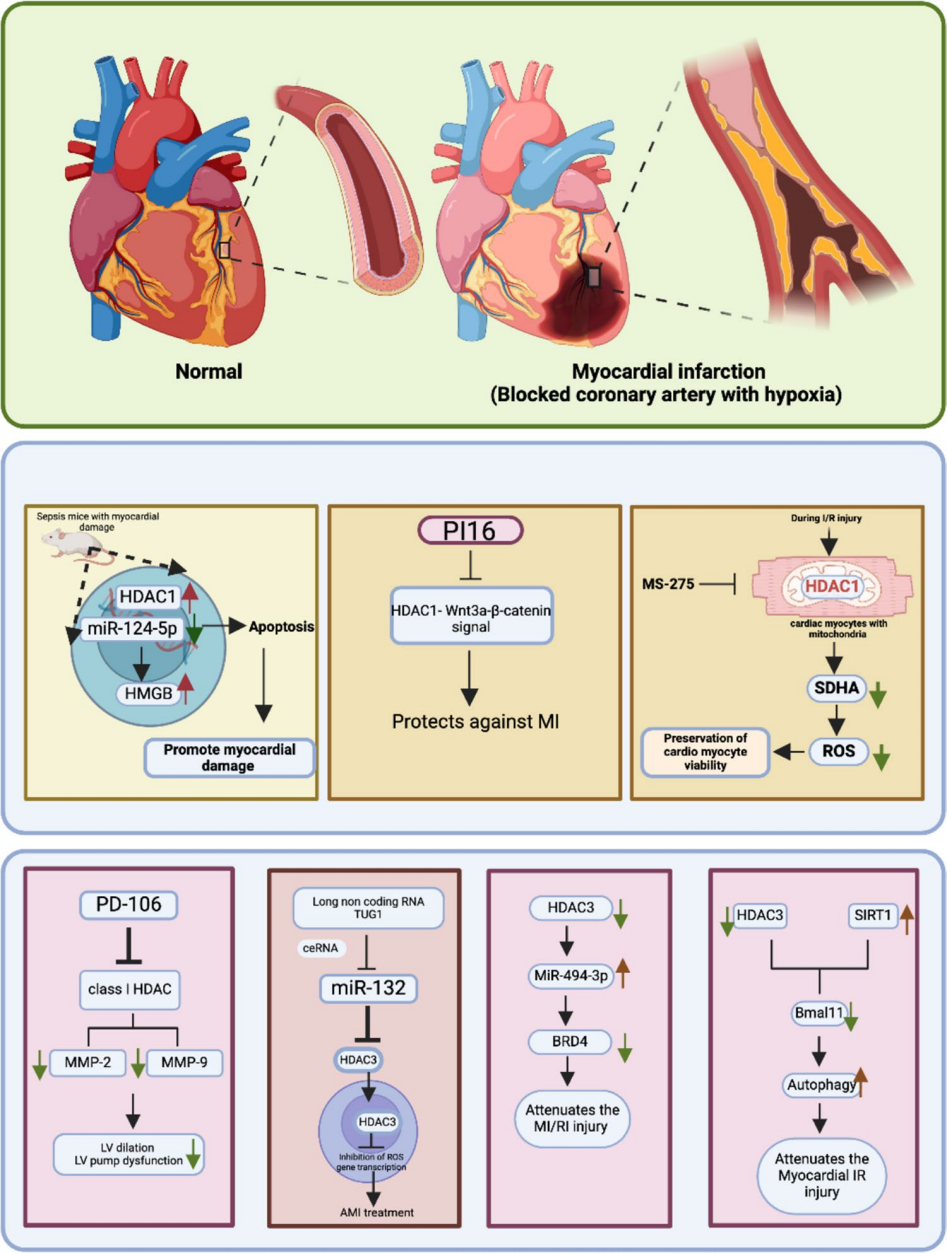
HDAC association with proteins and genes regulates myocardial infarction

Zheng and colleagues reported that mice with myocardial ischemia‒reperfusion injury (MI/RI) exhibit decreased miR-494-3p expression and increased HDAC3 and bromodomain-containing protein 4 (BRD4) expression. Increased levels of BRD4 shield the effect of miR-494-3p on MI/RI by limiting apoptosis and suppressing inflammation, suggesting its involvement in MI/RI. The miR-494-3p level was increased in the mimic group, whereas the BRD4 level was decreased even in the oe-BRD4 group. HDAC3 binds to the promoter region of miR-494-3p, indicating that its target is related to BRD4 [72]. Qiu et al. reported that myocardial IR injury is more prominent in diabetic rats than in nondiabetic rats. These cells exhibit elevated HDAC3 expression and attenuated SIRT1 levels, along with decreased BmaII and autophagy levels. The use of an HDAC3 inhibitor and SIRT1 agonist reduces injury by attenuating HDAC3 expression and improving SIRT1 activity, thus increasing the degree of autophagy induced by BmaI. Hypoxia/reoxygenation (H/R) stimulation increases high glucose (HG)-mediated reductions in cell viability, LDH levels, ROS production and reduces the mitochondrial membrane potential. Both low glucose (LG) and high glucose levels increase autophagic flux under H/R injury, but autophagic flux decreases in the LG+H/R group. HDAC3 levels are elevated, and SIRT1 and BmaI expression decreases under HG conditions stimulated by H/R. HDAC3 inhibition and SIRT1 activation alleviate HG+H/R-induced injury by increasing BmaI-like autophagy [73] (Table 5; Fig. 4).

**Table 5:** Genes, proteins, signalling cascades, and the implications of associated HDACs in myocardial infarction

Isoform	Protein/gene/Pathway involved	Histological and pathological inference	References
HDAC1	SDHA	HDAC1 localizes to the mitochondria of cardiac myocytes and contributes to early cardiac reperfusion injury MS-275 greatly reduces I/R injury, improving LV function and tissue viability by the end of reperfusion	[67]
	miR-124-5p, HMGB1	The levels of HMGB1, inhibited by the downregulation of HDAC1, are raised dramatically in the myocardium in case of severe sepsis	[68]
	PI16, Wnt3a/β- catenin pathway	HDAC1 inhibition by PI16 improves lLV remodelling and heart function after MI	[69]
HDAC3	MMP-2, MMP-9	MMP-2 and MMP-9 levels post-MI are reduced when treated with PD-106 LV dilation, pump dysfunction and activation of the MMP-9 gene promoter are reduced in mice treated with SAHA or TSA	[70]
	miR-132-3p, TUG1, Bcl-xL, Hsp70	Damage induced by H_2_O_2_ can be prevented using HDAC3 inhibitor RGFP966	[71]
	miR-494-3p, BRD4	Inhibited HDAC3 or elevated miR-494-3P plays a protective role in myocardial ischemia–reperfusion injury via suppression of BRD4	[72]
	SIRT1, BmaI	HDAC3 inhibition and SIRT1 activation alleviates injury induced by HG+H/R by elevating BmaI liked autophagy	[73]

In the context of MI, HDAC1 and HDAC3 are crucial as they foster inflammation, oxidative stress, and cardiomyocyte apoptosis. By reducing ROS and preserving tissue viability, the inhibition of HDAC1, especially in mitochondria—enhances cardiac recovery following I/R. Although HDAC1 contributes to myocardial damage during sepsis, PI16 protects the heart by downregulating both HDAC1 and the Wnt3a/β-catenin pathway. HDAC3 worsens post-MI remodelling via MMP activation and compromised autophagy, particularly in diabetes. Inhibiting these enzymes enhances histone acetylation, miRNA protection, and cardiac function, highlighting HDACs as promising therapeutic targets.

### Atherosclerosis

Hyperlipidaemia and lipid oxidation are the leading causes of atherosclerosis. Plaque formation affects the full vascular system to the coronary arteries from the aorta. Plaque formation is initiated by the deposition of cholesterol crystals in the intima and the underlying smooth muscles [74].

**Fig. 5:**
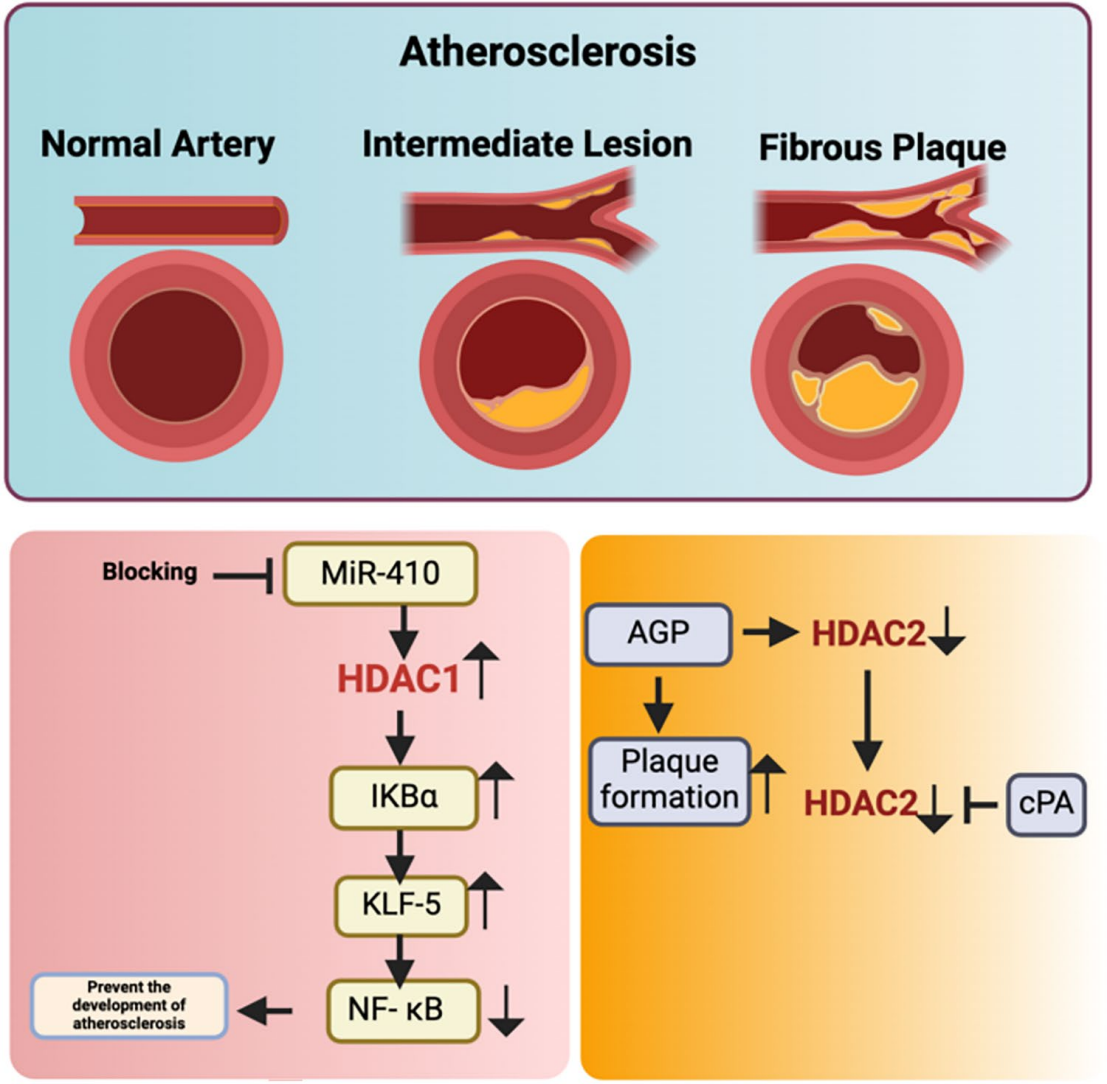
HDAC association with proteins and genes regulates atherosclerosis

Nan and colleagues identified miR-410 as a microRNA that targets HDAC1 in patients with atherosclerosis. Increased level of miR-410 in cholesterol exposed cells negatively affects HDAC1 expression by binding to the KLF5 transcription factor to suppress the transcription of the HDAC1 gene and to promote the expression of nuclear factor of kappa light polypeptide gene enhancer in B cells inhibitor alpha (IKBα); IKBα, whose degradation activates the NF-$\kappa$ B protein, which is involved in the inhibition of endothelial cell dysfunction, thus causing atherosclerosis. IKBα plays a major role in cell proliferation and the suppression of NF-κB, which predisposes cells to the development of atherosclerosis [75]. Tsukahara and colleagues emphasized alkyl-glycerophosphate (AGP) as a risk factor for a predisposition toward the formation of atherosclerotic plaques and endothelial cell inflammation. AGP downregulates HDAC2 in humans in endothelial of coronary artery (HCAECs) and is involved in the upregulation of endogenous inflammatory factors such as interleukin-6 and interleukin-8. Cyclic phosphatidic acid (cPA) inhibits this downregulation of HDAC2 in endothelial cells via the AGP, thus attenuating the effects of atherosclerosis [76] (Table 6) (Fig. 5).

**Table 6:** Genes, proteins, signalling cascades, and the implications of associated HDACs in atherosclerosis

Isoform	Protein/gene/Pathway involved	Histological and pathological inference	References
HDAC2	IL-6, IL-8, cPA	cPA inhibits the downregulation of HDAC2 in endothelial cells by AGP thus attenuating the effects of atherosclerosis	[75]
HDAC1	miR-410, IKBα, NF-kB	IKBα is involved in the cell proliferation and suppression of NF-kB which causes the predisposition toward the development of atherosclerosis	[76]

In summary miR-410 and alkyl-glycerophosphate (AGP) play a role in atherosclerosis by downregulating HDAC1 and HDAC2, leading to increased inflammation and endothelial dysfunction. In contrast, cyclic phosphatidic acid (cPA) mitigates the effects of AGP, providing protection against vascular inflammation.

### Cardiac fibrosis

The deposition of excess extracellular matrix (ECM) by cardiac fibroblasts leads to cardiac fibrosis (CF). It commonly occurs in MI, hypertensive heart disease, and other cardiomyopathies. Reactive interstitial fibrosis and replacement fibrosis are the most relevant classifications of cardiac fibrosis [77].

#### HDAC1

Lin and colleagues reported an association between HDAC1 and CFs. CFs secrete collagen and matrix metalloproteinases, which promote cardiac fibrosis in rats. HDAC1 is overexpressed in cardiac fibroblasts. In addition, ellagic acid was found to reduce the expression of HDAC1 in post-MI- and angiotensin-II-stimulated rat CFs. Along with reducing HDAC1, it also decreases MMP-9, MMP-2, collagen III and collagen I, expression. Since HDAC inhibitors are known to cause cell cycle arrest, the suppression of HDAC1 causes the division of cardiac fibroblasts to halt, thus attenuating the effect of fibrosis post-MI [78]. PI16 plays a major role in weakening cardiac fibrosis. Ang-II-induced cardiac fibroblast formation leads to the overexpression of HDAC1. PI16 overexpression reduces the Ang II-induced upregulation of HDAC1. A reduction in HDAC1 leads to an increase in the expression of p53, which shows that p53, a very important downstream gene, contributes to the attenuation of cardiac fibrosis by helping PI16 in cardiac fibroblasts [79].

#### HDAC3

**Fig. 6:**
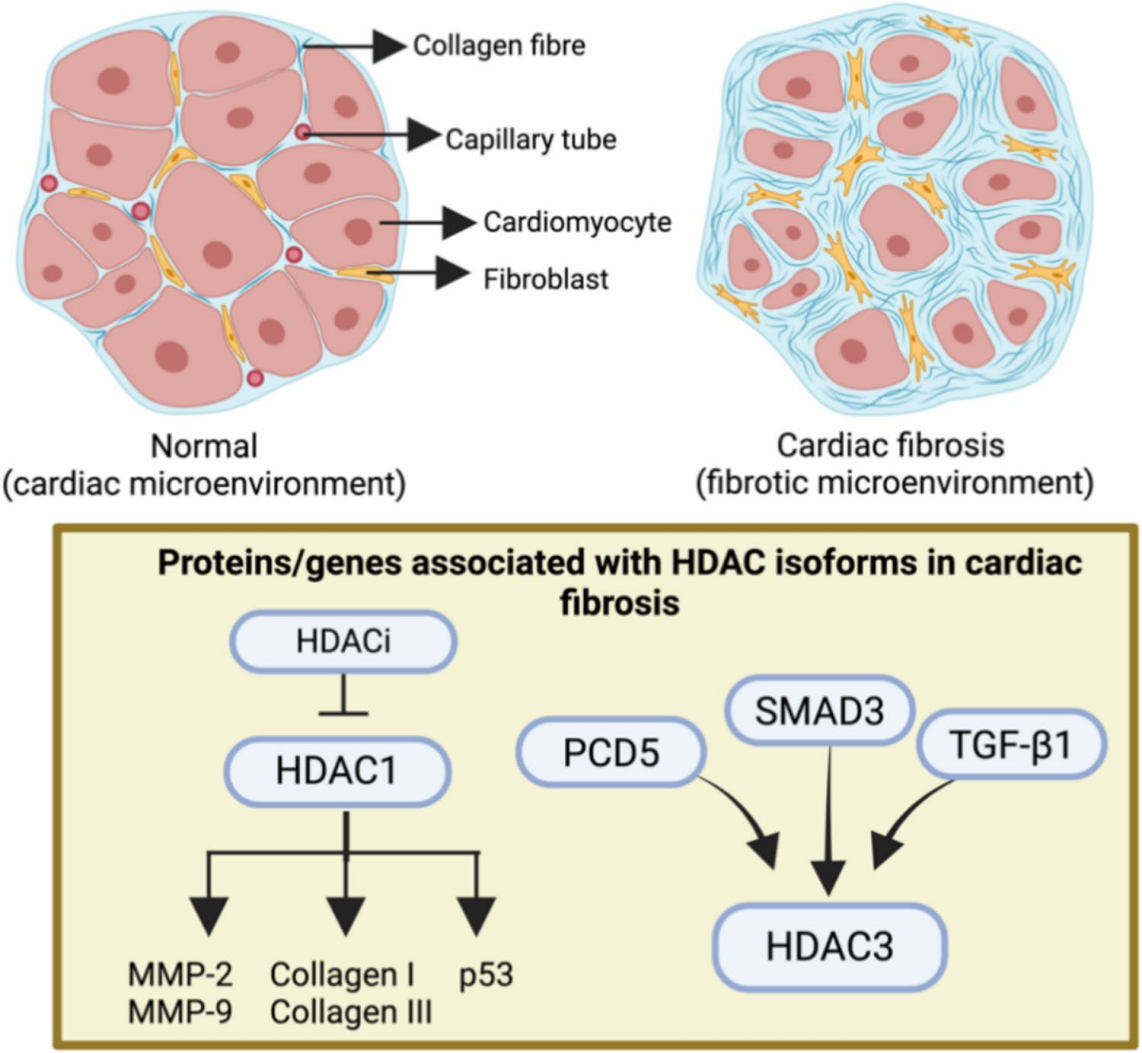
HDAC association with proteins and genes regulates cardiac fibrosis

Programmed cell death 5 (PCD5) is elevated in the cells of subjects with cardiac fibrosis. TGF-β1 induces an increase in the HDAC3 protein. An increase in the expression of PDCD5 stimulates TGF-β1 to reduce the expression of profibrogenic proteins and promote HDAC3 ubiquitination, thus inhibiting it and reducing fibrotic responses. In mothers, SMAD3 directly regulates PDCD5 in cardiac fibroblasts during fibrosis. PDCD5, in turn, helps prevent progressive fibrosis in the heart. SMAD3 is responsible for upregulating the expression of PDCD5 during cardiac fibrosis. Inhibition of HDAC3 blocks the fibrotic response induced by PDCD5 deficiency [80] (Fig. 6; Table 7).

**Table 7:** Genes, proteins, signalling cascades, and the implications of associated HDACs in cardiac fibrosis

Isoform	Protein/gene/Pathway involved	Histological and pathological inference	References
HDAC1	Angiotensin-II	Suppression of HDAC1 causes a division of cardiac fibroblasts thus attenuating the effect of fibrosis post-MI Ellagic acid reduces the expression of HDAC1 in post-MI- and angiotensin-II-stimulated rat CFs and decreases collagen I, collagen III, MMP-2 and MMP-9 expression	[78]
HDAC1	PI16, Ang-II, p53	p53 is an important downstream gene that contributes to the attenuation of cardiac fibrosis by helping the PI16 in cardiac fibroblasts	[79]
HDAC3	PDCD5, SMAD3, TGF-β1	Inhibition of HDAC3 blocks fibrotic response induced by PDCD5 deficiency	[80]

In summary HDAC1 and HDAC3 are crucial in advancing cardiac fibrosis through the activation of CFs and profibrotic signalling pathways. In CFs, HDAC1 is overexpressed and plays a role in the production of collagen and MMP; when it is inhibited by substances such as ellagic acid or PI16, fibrosis diminishes due to a reduction in CF proliferation and an elevation in p53 expression. In a like manner, during fibrosis, TGF-β1 induces an increase in HDAC3 levels, which correlates with a reduction in the expression of antifibrotic proteins. PDCD5, under the regulation of SMAD3, enhances the ubiquitination of HDAC3, which inhibits its profibrotic effects. Inhibiting either HDAC1 or HDAC3 effectively diminishes fibrotic responses in the heart.

### Myocarditis

Myocarditis refers to inflammation of the myocardium that may occur after inflammation due to exposure to toxic substances or possibly immune-mediated damage. There are different forms of myocarditis, such as acute, fulminant, subacute and chronic. Approximately 30% of the population with myocarditis develops cardiomyopathy [81].

**Fig. 7:**
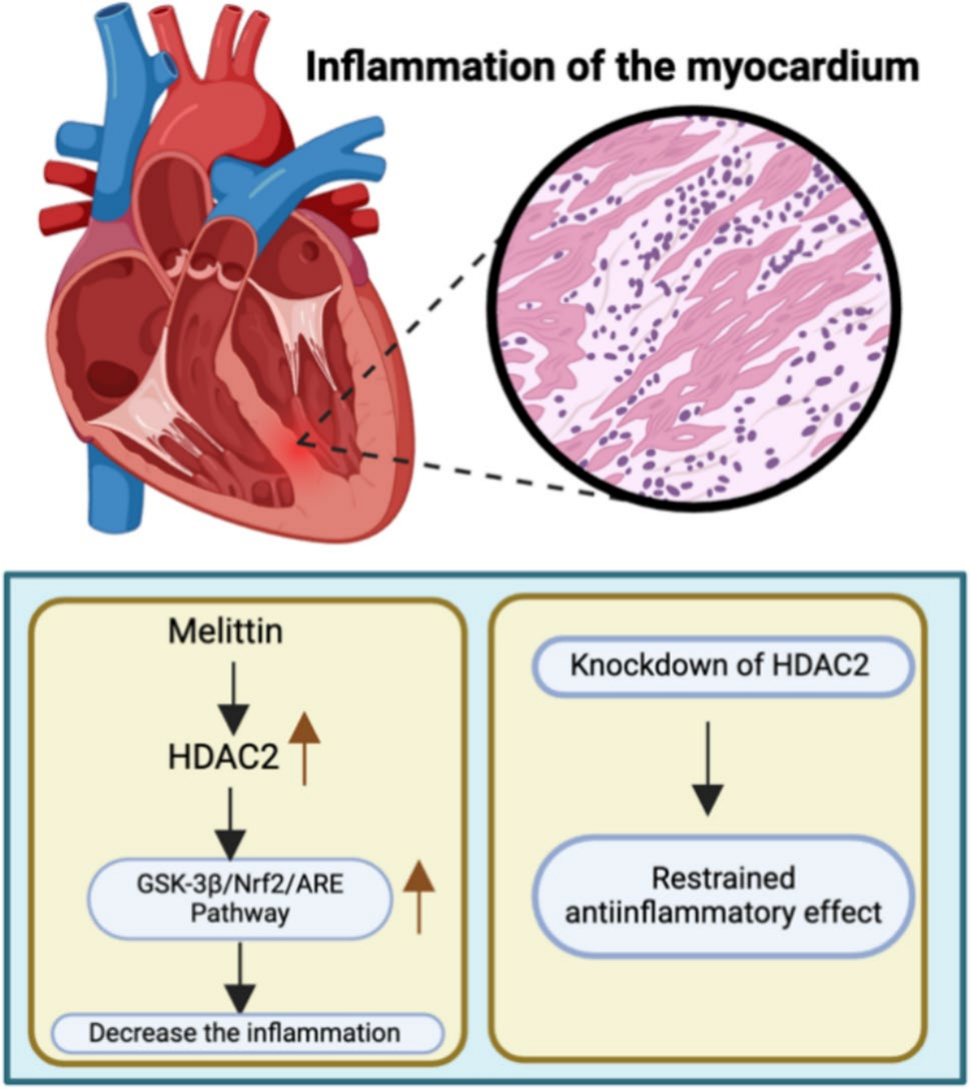
HDAC association with proteins and genes regulates myocarditis

Wang and group showed that myocarditis is caused by heart muscle damage and inflammation caused by several chemicals. Viral myocarditis (VMC) can be induced by Cox-sackievirus (CVB3) in mice. These mutants are then treated with melittin, a polypeptide of honeybee venom that has several applications, including in inflammation treatment. Melittin is known to ameliorate myocardial injury in VMC mice. The Nrf2/ARE pathway and the GSK-3β pathway upstream of this pathway were activated by the upregulation of HDAC2. Both the pathway and GSK-3β are involved in anti-inflammatory effects in cardiac cells. Thus, activation of the GSK-3β/Nrf2/ARE signalling pathway is known to reverse the effects caused by CVB3 [82]. Zhang et al. reported that myocardial inflammation occurs in heart muscle due to several diseases. Lipopolysaccharide (LPS) is one such cause of inflammation. Glucocorticoids (GCs) are known to reverse these effects. All these changes occur in the fibroblasts of the heart. LPS activated several inflammatory genes, such as TNF-α and IL-1β. Oxidative stress caused by xanthine oxidase/xanthine caused HDAC2 expression knockdown that further prevented GCs to act adequately in restraining the anti-inflammatory response. Hydrocortisone was found to increase the levels of HDAC2 in response to LPS-induced inflammation. However, HDAC2 knockout reduced these effects [83] (Fig. 7; Table 8).

**Table 8:** Genes, proteins, signalling cascades, and the implications of associated HDACs in myocarditis

Isoform	Protein/gene/Pathway involved	Histological and pathological inference	References
HDAC2	Nrf2/ARE pathway and GSK3β	Activation of the GSK3β/Nrf2/ARE signalling pathway is known to reverse the effects caused by CVB3	[82]
HDAC2	TNF-α and IL-1β	HDAC2 is required by the physiological concentration of glucocorticoid to inhibit inflammation in cardiac fibroblasts	[83]

In summary, myocarditis protection by HDAC2 occurs through the activation of anti-inflammatory pathways. In cases of inflammation caused by viruses and LPS, the increase of HDAC2 through melittin or glucocorticoids leads to a decrease in myocardial damage; conversely, when HDAC2 is downregulated, the anti-inflammatory response is weakened.

### Atrial fibrillation

Abnormal firing of electrical impulses in the atria, which override the natural pacemaker of the heart, is a characteristic feature of atrial fibrillation. Atrial fibrillation causes irregular contractions of the atrial muscles, leading to symptoms such as palpitations, shortness of breath, and an irregular heart rate [8].

Zhang and group investigated the role of class I and IIa HDACs in atrial fibrillation (AF), a common cardiac arrhythmia associated with altered gene transcription. This research aimed to understand how these HDACs contribute to contractile dysfunction in AF and their potential as therapeutic targets. The overexpression of HDAC1 and HDAC3 depleted the calcium transient (CaT) amplitude in cardiomyocytes, both under normal pacing conditions and more significantly during tachypacing. However, CaT is not affected by the overexpression of class IIa HDACs in normal cardiomyocytes, as HDAC7 and HDAC5 even shield against the tachypacing-induced CaT reduction. When dominant negative mutants of these HDACs were upregulated in cardiomyocytes, the shielding effect of HDAC5 and HDAC7 was diminished. When compared with those from patients with sinus rhythm, the atrial tissue from AF patients showed increased expression levels and fetal gene expression, activity of HDAC3 and phosphorylated HDAC5 [84].

In summary, although HDAC1 and HDAC3 are involved in the development of calcium management defects associated with atrial fibrillation, class IIa HDACs such as HDAC5 and HDAC7 provide protection from dysfunction caused by tachypacing.

### HDAC isoforms associated with other cardiovascular disorders

#### HDAC1

Multiple individuals in the USA are affected by left ventricular heart failure (LVHF), with new cases emerging every day. The interaction between thrombospondin 1 (TSP1) and CD47 promotes cell injury by inhibiting the NO signalling pathway. LVHF is characterized by increased TSP1 expression, and the loss of CD47 protects against LVHF [85]. Blakeslee and colleagues elucidated sumoylation as a posttranslational modification that adds an ubiquitin-like structure to the target gene. The inhibition of HDAC class I, specifically HDAC1/2 or HDAC2 alone, leads to an increase in SUMOylation due to the accumulation of small ubiquitin related modifier –1 (SUMO-1)-conjugated proteins in cardiomyocytes. Lysine residues have different effects on SUMOylation and acetylation, such as in the p53 gene, which causes its activation or deactivation. The activation of this gene due to SUMOylation is the reason for the protective effect of HDAC inhibitors on cardiomyocytes and fibroblasts [86]. Cardiovascular and metabolic diseases are associated with vascular calcification (VC). Decreases in HDAC1 activity via either chemical inhibitors or genetic ablation enhance VC. In human calcified coronary artery and animal calcification models, a reduction in HDAC1 protein but not in mRNA is observed. VC is preceded by HDAC1 under calcification-inducing conditions, which is mediated by the mouse double minute 2 homologue (MDM2) E3 ubiquitin ligase that begins HDAC1 K74 ubiquitination and proteasomal degradation of HDAC1. Downregulation of MDM2 blunts VC, whereas upregulation of MDM2 intensifies VC. Decoy peptides spanning MDM2, RG 7112 inhibitor, and HDAC1 K74 prevent VC in vivo and in vitro. These results point towards the role of MDM2-mediated ubiquitination of HDAC1 as a new therapeutic target in VC [87].

Li and the group showed that myocardial oedema is the increased water content in the myocardium of the heart. The role of HDAC1 in hypoxia-induced H9c2 cardiac cell swelling was investigated. Induced hypoxia or chemical hypoxia results in increased HDAC1 expression and decreased HDAC4 expression. Inhibition of HDAC1 improved these conditions by decreasing cell swelling. HDAC1 inhibition improved cell viability and decreased cell volume, cell death, and lactate dehydrogenase leakage. Chemical hypoxia led to an increase in the granularity on the surface of swollen H9c2 cells regardless of HDAC1 inhibition. HDAC1 regulates intercellular osmotic pressure, thus attenuating the effects of cell swelling. HDAC1 downregulation increases the Young’s modulus of the cell, thus reversing the effects of myocardial oedema [88]. HDAC1 is overexpressed in patients with TAD. Downregulation of HDAC1 upregulates smooth muscle 22 α (SM22α) and α‐smooth muscle actin (α‐SMA). In addition, vascular smooth muscle cells (VSMCs) show an increased ability to survive and migrate. PKD1 negatively regulates HDAC1 expression and is responsible for the increased expression of SM22α and α‐SMA. The upregulation of PKD1 leads to the suppression of VSMC viability and migration by upregulating contractile phenotype markers, which are suppressed by HDAC1 upregulation in patients with transverse aortic dissection (TAD) [89]. TAD involves separation of the layers of the aorta caused by degeneration of the aortic media. TADs have a high mortality rate, with a low occurrence rate. Rapid detection and treatment methods have enhanced the outlook of TAD patients, and new diagnostic modalities are being developed to allow prompt detection [90].

#### HDAC3

Sun and group showed that HDAC3, which is deactivated in the cardiac and skeletal muscles of mice postnatally, remains active for more than a year in mice fed normal chow. These patients develop heart failure and hypertrophic cardiomyopathy when they are switched to a high-fat diet. HDAC3^fl/fl^/MCK-Cre mice (MCH3-KO). By the end of 4 months, the heart weight-to-tibia length ratio increased, indicating the onset of cardiac hypertrophy. Mild fatty acid infiltration was observed by the end of 8 months in the hearts of MCH3-KO mice. The hearts had better systolic function, the skeletal muscle had normal grip strength and weight, and survival was not reduced when they were fed normal chow. The downregulated genes were enriched in mitochondrial bioenergetics processes and lipid metabolism in the myocardium of MCH3-KO mice. The genes related to the immune system were enriched when upregulated in the myocardium. A high-fat diet (HFD) is lethal to MCH3-KO mice and elevates ANP and BNP expression, indicating heart failure. Widespread fibrosis is observed across the ventricles, and the cardiomyocyte diameter increases, indicating myocyte hypertrophy. These results show that HDAC3 loss in cardiac tissue under a HFD causes heart failure and lethality [91]. VPA, a class I HDAC inhibitor, disrupts planar cell polarity (PCP) signalling in heart cells, leading to congenital heart defects (CHD) and cardiac teratogenesis. Pregnant mice treated with VPA showed smaller hearts with thin ventricular walls, compact layer patches, and deep intratrabecular recesses compared with controls. These abnormalities were linked to the suppression of key PCP genes *Vangl2* and *Scrib*, while *Rac1* remained unaffected. VPA also reduced *Hdac* expression in embryonic hearts, with specific *Hdac3* repression causing PCP gene disruptions. *Hdac3* overexpression reversed these effects, mitigating VPA-induced cardiac abnormalities [92]. Fibroblast growth factor 21 (FGF21), a regulator of lipid and glucose metabolism, protects against diabetes-induced cardiovascular injuries but is inhibited by HDAC3. FGF21 levels were significantly reduced in diabetic mice, and HDAC3-specific inhibition (via RGFP-966) increased FGF21 more effectively than valproic acid (VPA). HDAC inhibitors downregulated Keap1, enhancing Nrf2, an anti-inflammatory factor, through miRNA-200a-mediated suppression of Keap1 translation. Specific HDAC3 inhibition reduced Keap1, CTGF, and TNF-α levels, decreasing vascular thickening and oxidative stress in diabetic mice. RGFP-966 and VPA also improved antioxidant gene expression, with RGFP-966 increasing CAT, NAD(P)H quinone oxidoreductase 1 (NQO1), and heme-oxygenase-1 (HO-1) mRNA levels. HDAC3 inhibition demonstrated superior benefits in mitigating diabetes-associated cardiovascular damage [93].

#### Shared mechanistic roles of multiple HDAC isoforms

Nural–Guvener and the group showed that CHF caused by plaque accumulation is reduced by the HDAC class I inhibitor Mocetinostat. Interleukin-6 (IL-6), a proinflammatory cytokine, is an important marker for CHF. Mocetinostat works by inhibiting the expression of this protein, which is involved in the activation of the JAK/signal transducers and activators of transcription (STAT) pathway, resulting in the production of the STAT3 protein. STAT3 is a prominent fibrotic activator in the heart. STAT3 is activated in the left ventricle in CHF patients through its phosphorylation at Y705, which is directly involved in heart failure. STAT3 inhibition is beneficial because it causes a decrease in fibrotic processes in the heart [94]. Lkhagva and colleagues studied the role of HDACs in TNF-α-induced mitochondrial dysfunction in heart cells and potential therapeutic strategies and underlying mechanisms. This research assessed mitochondrial function in HL-1 cells exposed to TNF-α with and without HDAC inhibition. This study revealed that TNF-α upregulates the activity of Class I and Class II HDACs (excluding Class IIa) and increases the expression of Class I HDACs (HDAC1, HDAC2, HDAC3, and HDAC8) in HL-1 cells. TNF-α also leads to mitochondrial dysfunction characterized by reduced basal and ATP-linked respiration, increased mitochondrial superoxide production and decreased cellular ATP production. Importantly, this dysfunction is attenuated by downregulating HDACs using compounds such as MPT0E014 or MS-275. Inhibiting HDACs could be a therapeutic approach to address mitochondrial dysfunction and oxidative stress induced by TNF-α in cardiomyocytes, potentially offering a strategy to mitigate cardiac dysfunction [95] (Table 9).

**Table 9:** Genes, proteins, signalling cascades, and the implications of associated HDACs with different cardiac conditions

Isoform	Protein/gene/Pathway involved	Histological and pathological inference	References
HDAC1	TSP1 and CD47, NO signalling pathway	The activation of p53 gene due to SUMOylation is the reason for the protective role by HDAC inhibitors cardiomyocytes and fibroblasts	Blakeslee et al. [86]
	MDM2	MDM2-mediated ubiquitination of HDAC1 is a new therapeutic target in VC	[87]
	-	HDAC1 downregulation increases the Young’s modulus of the cell thus reversing the effects of myocardial oedema	[88]
	α‐SMA, SM22α, PKD1	Upregulation of PKD1 leads to suppression of the VSMC viability and migration	[89]
HDAC3	-	HDAC3 loss in the cardiac tissue under an HFD causes heart failure and lethality	[91]
	PCP signalling, Vangl2 and Scrib mRNA	Disruption of planar cell polarity pathway attributable to valproic acid-induced congenital heart disease through HDAC3 participation in mice	[92]
	FGF21, Keap1. Nrf2	HDAC3-specific inhibition showed a better effect on oxidative stress-responsive gene mRNA levels RGFP-966 and VPA improves oxidative stress-responsive gene mRNA levels	[93]
Multiple HDAC isoforms	IL-6, JAK/STAT pathway, Y705	Inhibition of STAT3 is beneficial as it causes a decrease in the fibrotic processes in the heart Mocetinostat works by inhibiting the expression of IL-6 protein, which is involved in the activation of the JAK/STAT pathway	[94]
	TNF-α	Inhibiting HDACs could be a therapeutic approach to address mitochondrial dysfunction and oxidative stress Using MPT0E014, MS-275 could be a therapeutic approach to address mitochondrial dysfunction and oxidative stress induced by TNF-α in cardiomyocytes,	[95]

In summary HDAC1 and HDAC3 are crucial in cardiovascular diseases. HDAC1 plays a role in vascular calcification, myocardial oedema, and TAD, but its inhibition provides protective benefits. Under high-fat diets, the absence of HDAC3 leads to heart failure and is associated with congenital defects and damage to the heart in diabetes. By improving mitochondrial function, reducing inflammation, and lowering fibrosis, HDAC inhibition shows promise as a therapeutic approach for heart conditions.

## Discussion and future prospects

HDAC1 and HDAC2 are the most extensively studied isoforms in cardiovascular research. They are known to play pivotal roles in cardiac development, remodelling, and disease. These isoforms are involved in regulating inflammation, hypertrophy, and fibrosis, which are key processes in the progression of CVDs. For instance, HDAC2 has been identified as crucial for reducing inflammation in myocarditis, a condition characterized by inflammation of the heart muscle. The inhibition of HDAC2 activity has shown potential for attenuating inflammatory responses, suggesting that HDAC2 is a therapeutic target for reducing cardiac inflammation. HDAC3 has also been implicated in cardiovascular pathology, particularly in the context of fibrosis. SMAD3, a transcription factor involved in fibrotic responses, is known to directly regulate PDCD5 in cardiac fibroblasts; thus, PDCD5 inhibits HDAC3. This inhibition of HDAC3 is essential for preventing progressive fibrosis in the heart, highlighting the intricate regulatory mechanisms involving HDAC3 in cardiac fibrosis. In contrast to HDAC1 and HDAC2, HDAC8 has received comparatively less attention in cardiovascular research. Few studies have explored its role in cardiac remodelling and disease, leaving a gap in understanding its specific functions and potential as a therapeutic target. The limited knowledge about HDAC8 underscores the need for more focused research to elucidate its role in CVDs.

In conclusion, HDAC isoforms play diverse and critical roles in cardiovascular diseases. Although HDAC1 and HDAC2 have been well characterized, there remains a significant knowledge gap regarding the functions of other isoforms, such as HDAC8. Further research into these less-studied isoforms is essential to fully understand their roles and to harness their potential in developing targeted therapies for CVD treatment.

## Data Availability

No datasets were generated or analysed during the current study.
